# Recent Advances in Polysaccharide-Based Encapsulation of Probiotics: Encapsulation Technologies, Material Properties, and Applications in Dairy Products

**DOI:** 10.3390/foods15172969

**Published:** 2026-08-24

**Authors:** Jinyu Ma, Mengying Zhang, Huifang Lan, Wanyi Chen, Jiage Ma

**Affiliations:** Key Laboratory of Dairy Science, Ministry of Education, College of Food Science, Northeast Agricultural University, Harbin 150030, China; majinyu0133@163.com (J.M.); zhangmengying806@163.com (M.Z.); 13488574730@163.com (H.L.); wanyic602@gmail.com (W.C.)

**Keywords:** encapsulation, probiotic, polysaccharide, dairy

## Abstract

Probiotics have received widespread attention due to their beneficial effects on human health. However, ensuring their viability and functionality during processing, storage, and gastrointestinal transit can be challenging. Encapsulation technology represents a key strategy to address these issues. Among available encapsulation technologies, polysaccharide-based systems have garnered growing interest. This review provides a detailed overview of the characteristics and applications of various polysaccharides, as well as representative strategies for their modification. In addition, the formulation of polysaccharide-based composite wall materials combined with additional polysaccharides, proteins, or metal ions is discussed as an effective strategy for enhancing the stability of encapsulated probiotics Encapsulation techniques, including extrusion, emulsification, spray drying, freeze drying, electrospraying, electrospinning, and layer-by-layer assembly, are critical for probiotic viability, storage stability and release behavior. The review also considers emerging approaches, such as 3D bioprinting and microfluidics, and applications in dairy products, including impacts on product quality and functional properties. Overall, the review describes the application potential of polysaccharide-based encapsulation and future research directions for dairy products and other functional foods.

## 1. Introduction

Probiotics are defined by the Food and Agriculture Organization of the United Nations/World Health Organization (FAO/WHO, 2002) as live microorganisms that confer a health benefit to the host when administered in adequate amounts [1]. They can regulate and enhance immune responses, maintain intestinal microbiota homeostasis, and modulate the nervous system [2,3]. However, the survival and functional characteristics of probiotics are affected by several different factors [4]. For example, their viability may decline by 10–30% due to metabolite accumulation during manufacturing [5]. During storage, temperature fluctuations can lead to ice crystal formation and changes in water activity, further impairing viability [6]. Following oral ingestion, the strongly acidic gastric environment, along with digestive enzymes and bile salts in intestinal fluid, inactivates most probiotic cells and restricts their colonization and proliferation within the gastrointestinal tract [7]. FAO/WHO guidelines recommend that probiotic foods contain a minimum of 10^6^ CFU/g of viable microorganisms, although this level is not a fixed universal requirement and may vary with probiotic strain, product formulation, intended health benefit, and applicable regulatory frameworks [8]. Therefore, developing effective strategies to ensure that probiotics remain active until they reach the host’s gut has gained significant research interest [9].

Encapsulation technology can be regarded as the primary method for improving the survival ability of probiotics [10]. This technology protects probiotics from damage through various methods such as forming a protective barrier around the probiotic cells [11,12]. The type of encapsulating materials has been demonstrated to have a direct impact on the survival of probiotics [13]. Available wall materials including carbohydrates, proteins, and lipids [13,14]. Among the various available encapsulation substrates, polysaccharides are widely recognized to have significant potential for use in probiotic encapsulation due to their abundance, high food safety standards, and excellent biocompatibility [15,16]. Polysaccharides currently used for probiotic encapsulation include sodium alginate, pectin, gellan gum, xanthan gum, starch, and cellulose [17], among which sodium alginate has become the most widely used wall material for probiotic encapsulation due to its mild crosslinking conditions, good biocompatibility, and cost-effectiveness [18]. However, single polysaccharide wall materials also have limitations, including high porosity, insufficient mechanical strength, and weak acid resistance [17]. Recent attempts to develop polysaccharide-based composite encapsulation systems including pectin–sodium alginate and alginate–chitosan, which form double-layer and multi-layer composite microcapsules [19,20,21]. Encapsulation efficiency and gastrointestinal survival can be significantly improved through the synergistic effects achieved when combining different polysaccharides [22,23,24].

**Figure 1:**
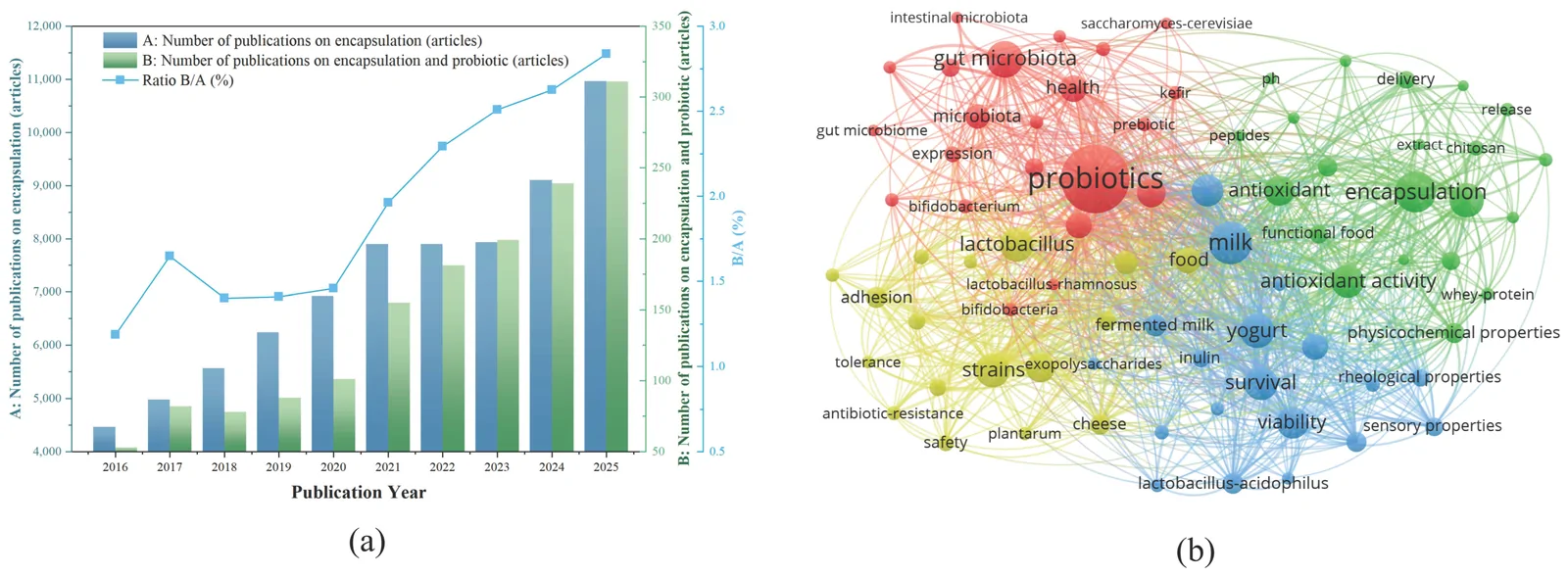
(**a**) Number of articles in the Web of Science database with the keywords “encapsulation” and “probiotic”. The single-term search TS = (encapsulation) retrieved 71,970 records after removing duplicates and limiting document types to articles and review articles; the combined search TS = (encapsulation) AND (probiotic) retrieved 1487 records after the same screening process; access date: 10 August 2026. (**b**) Keyword co-occurrence network map generated using the VOSviewer (1.6.20) software. TS = (probiotic OR encapsulation) AND Dairy; time span: 2021–2025; document types: articles and reviews; access date: 11 August 2026. The analysis was performed using VOSviewer with a minimum occurrence threshold of 40.

Dairy products include fluid milk, fermented milk, cheese, and milk powder, which are derived from cow’s milk, goat’s milk, and other commonly used mammalian sources [25]. It is widely acknowledged that milk and dairy products are a rich source of essential nutrients. These products also contain bioactive nutrients that assist in maintaining bodily functions [26]. Dairy products are among the most significant delivery vehicles for probiotics [27]. As one of the most important categories of functional foods, probiotic dairy products account for over 40% of the functional food market and play an indispensable role in promoting public health [28,29]. Nevertheless, probiotics in dairy products still suffer from decreases in viability. Changes in ambient temperature, pH, light exposure, and other environmental factors may reduce strain activity, impairing the functional performance and shelf life of the product [30,31]. Encapsulation technology has been proven to enhance the survival stability of probiotics in dairy matrices. Accordingly, encapsulation technology is widely recognized in industrial dairy product development. For instance, the annual number of publications on encapsulation and probiotics in the Web of Science database increased consistently from 2016 to 2025 (Figure 1a). Based on this database, this study uses the VOSviewer (1.6.20) software to conduct a bibliometric analysis of the literature on polysaccharide-based encapsulation technology over the past five years. The keyword co-occurrence network revealed four major clusters related to polysaccharide-based probiotic encapsulation and relevant applications in dairy products (Figure 1b). The red cluster, which includes “probiotics”, “gut microbiota”, and “health”, focuses on the functional properties and application value of probiotics. The green cluster, which includes “encapsulation”, “chitosan”, “delivery”, and “release”, emphasizes the development of polysaccharide-based encapsulation systems, including wall material selection and controlled release strategies for improving probiotic stability. The blue cluster, which includes “milk”, “yogurt”, “survival”, and “viability”, reflects the application of encapsulated probiotics in dairy products and stability and product quality evaluation. The yellow cluster, which includes “strains”, “adhesion”, and “tolerance”, represents research on probiotic strain characteristics and functional properties related to encapsulation performance. This article reviews and discusses recent research on polysaccharide-based encapsulation technology for probiotic encapsulation, including the polysaccharide wall materials used their structural features, and their effects on probiotics. The review also synthesizes the basic principles and process characteristics of several polysaccharide-based encapsulation technologies, such as extrusion, spray drying, and freeze drying. The current application status of polysaccharide-based probiotic encapsulation technology in dairy products is also considered, as well as challenges and future development trends.

## 2. Polysaccharide Wall Material

### 2.1. Sodium Alginate

Sodium alginate (SA) is a naturally occurring anionic polysaccharide, typically derived from brown algae or bacterial sources. Its macromolecular backbone comprises β-D-mannuronic acid (M) and α-L-guluronic acid (G), linked by (1→4) glycosidic bonds [32]. Given its exceptional qualities, including biosafety, biocompatibility, and renewability, it is used in many industries, including in the food sector [33]. The physical characteristics, biocompatibility, and functional performance of sodium alginate hydrogels are determined by the M/G ratio and the block sequences of G and M residues, which vary between alginates [34,35]. High-G alginate capsules are denser and adhere effectively to abdominal organs [36] while alginates with lower M/G ratios have been shown by Zhao et al. [37] to generate compact hydrogel networks with improved structural stability. The viability of encapsulated probiotics is directly affected by the M/G ratio. As such, high-G alginate is more suitable for encapsulating probiotics. Alginate can also react with divalent cations to form rigid gels and insoluble polymeric networks. The cross-linking effect is strongest when calcium ions bind to guluronic acid segments on the alginate macromolecular backbone and form the typical “egg-box” structure [38]. As Tordi et al. found [39], a wide range of cations beyond Ca^2+^ can complex with alginate, including monovalent, divalent, trivalent, and tetravalent ions such as Sr^2+^, Ba^2+^, Fe^3+^, Al^3+^, and Zr^4+^, and each cation uniquely influences the material properties through distinct coordination modes, enabling precise tailoring of the gel network structure and physicochemical performance.

Its excellent oxygen barrier properties and favorable release profile in simulated gastrointestinal fluid (SGF) make SA a promising encapsulating material for probiotics [40]. Hydrogel formed by alginate and calcium ions possesses pH-sensitive swelling ability. In gastric acidic conditions, the gel’s carboxyl group will be protonated, resulting in shrinkage and densification due to hydrogen bonding and ionic interactions, which confers acid resistance to the hydrogel and protects the core material from gastric acid degradation. In the neutral intestinal environment, carboxyl group deprotonation causes the gel to swell, triggering the release of its encapsulated cargo [41]. However, the high porosity and poor mechanical strength of alginate gels compromise their encapsulation efficiency, leading to premature release of the encapsulated substance. These disadvantages reduce the survival of encapsulated probiotics during gastrointestinal digestion and restrict the industrial translation of SA gels [42]. To address these issues, SA is frequently combined with other high-molecular-weight polymers to boost probiotic tolerance to unfavorable environmental conditions [43]. Common methods include polysaccharide integration, polysaccharide-protein complexation, and polysaccharide-metal ion chelation. Chitosan is a natural polycationic polymer that can form polyelectrolyte complexes with alginate and pectin through electrostatic association. As such, chitosan can be used as a coating material to provide a protective barrier over polysaccharide-based carriers. Furthermore, chitosan’s mucoadhesive and antimicrobial properties can provide further protection to composite carriers for encapsulated probiotics [44]. Hu et al. [45] created a double-layer microgel system that encapsulated *Lacticaseibacillus rhamnosus* GG. The outer layer included sodium alginate and chitosan for colon-targeted administration, while the inner layer included guar gum and low methoxyl pectin for colonic retention and breakdown. In vitro, this approach demonstrated good bioresponsive properties across a broad pH range, enabling site-specific release in the gastrointestinal tract and promoting stable probiotic colonization.

### 2.2. Pectin

Pectin is a naturally occurring anionic macromolecular polysaccharide located in the primary cell wall and intercellular layers of plant cells. It is mostly obtained from raw materials such as citrus peels, apples, and sunflowers [46]. This class of native heteropolysaccharides includes up to 17 monosaccharides and over 20 types of glycosidic linkages [47]. Structurally, pectins consist mainly of D-galacturonic acid (GalA) units connected via α-(1→4) glycosidic bonds [48]. These residues exhibit varying degrees of methylation and are organized into three principal domains: homogalacturonan, rhamnogalacturonan I, and rhamnogalacturonan II [47]. Based on their degree of esterification, pectins are categorized into low-methoxyl pectins (LMPs) and high-methoxyl pectins (HMPs) [49]. Xu et al. [50] demonstrated that HMPs exhibit inferior gel-forming capacity and greater pH sensitivity in contrast to LMPs. Furthermore, LMPs have better sustained-release qualities and enhances the targeted distribution of probiotics in the gastrointestinal tract (GIT).

Pectin is widely use in probiotic encapsulation due to its gelling qualities, biocompatibility, and non-toxicity [51]. It also forms hydrogels that create a strong barrier for encapsulated probiotics when calcium ions are added [52]. Both hydrophobic interactions and hydrogen bonds between methyl groups are necessary for high-methoxy pectin gelation. Low-methoxy pectin gelation produces the well-known “egg-box” structure through electrostatic interactions between free carboxylate groups and divalent cations such as calcium [50]. Small changes in how the chains are arranged in relation to one another within this framework results in distinct cross-linking structures, known as the “shifted egg-box.” Consequently, pectin beads exhibit excellent resilience, hardness, and encapsulation efficiency [53]. Their film-forming properties also enable them to create stable microcapsules [54]. However, pectin is also characterized by disadvantages in probiotic encapsulation, including high water solubility and a porous structure that makes it difficult to deliver probiotics to specific locations. Its poor thermal stability also compromises its suitability for long-term storage [55]. To protect probiotics and enhance encapsulation efficiency and long-term stability, pectin-based carriers can be blended with other encapsulating ingredients. In this context, protein–pectin composite carriers are highly promising. Proteins and pectin form composite carriers such as microcapsules, nanoparticles, and gels through non-covalent interactions, including electrostatic attraction and hydrogen bonding. Given their stronger intermolecular forced and emulsifying properties, compared to pectin carriers alone, protein–pectin composite carriers can withstand adverse conditions during processing and in the gastrointestinal tract, thereby enhancing the encapsulation efficiency, storage stability, and controlled release performance of probiotics [56]. To encapsulate *Lactiplantibacillus paraplantarum* LR-1, Rao et al. [57] created a composite material based on sodium alginate, pectin, and whey protein isolate as wall components. Along with greatly improved probiotic viability and heat resistance, the resultant material demonstrated good mechanical strength, controlled release characteristics, and storage stability. Zhang et al. [58] developed a pH-responsive microgel system that encapsulated *Heyndrickxia coagulans* BC99 and procyanidins via a microemulsion using pectin, whey protein isolate, and Ca^2+^ as wall materials, which effectively enhanced probiotic survival and stress tolerance.

### 2.3. Gellan Gum

Gellan gum (GG) is a natural anionic polysaccharide with gel-forming properties, and it is produced by *Sphingomonas elodea* and *Sphingomonas paucimobilis* [59,60]. Its backbone consists of a linear tetrasaccharide repeating unit made up of 1,3-β-D-glucose, 1,4-β-D-glucuronic acid, 1,4-β-D-glucose, and 1,4-α-L-rhamnose at equimolar ratios [59]. GG is one of the most commonly used biopolymers for probiotic microencapsulation [61]. It is a typical gelling polysaccharide that forms gels upon cooling. As the temperature decreases, chains change from a random coil configuration to a double helix state, followed by helix aggregation and network formation. Moreover, divalent cations promote GG gelation through double helix aggregation and intermolecular cross-linking, and they reinforce the helical structure and expedite network development [62]. GG has excellent gelling capacity, flexibility, biocompatibility, degradability, thermal stability, and acid resistance, as well as a versatile texture. These properties enable it to effectively protect probiotics against adverse conditions [63]. It should be noted that cross-linking agents include both food-grade and non-food-grade types, such as chemical cross-linkers. For food applications, only food-grade cross-linking strategies should be used. Gellan-gum-based gels are also stable under low-pH conditions. The characteristics of these gels depend on the cation type and concentration, pH, and temperature at the time of gelation, and the resulting gels are resistant to rapid enzymatic degradation [64].

**Figure 2:**
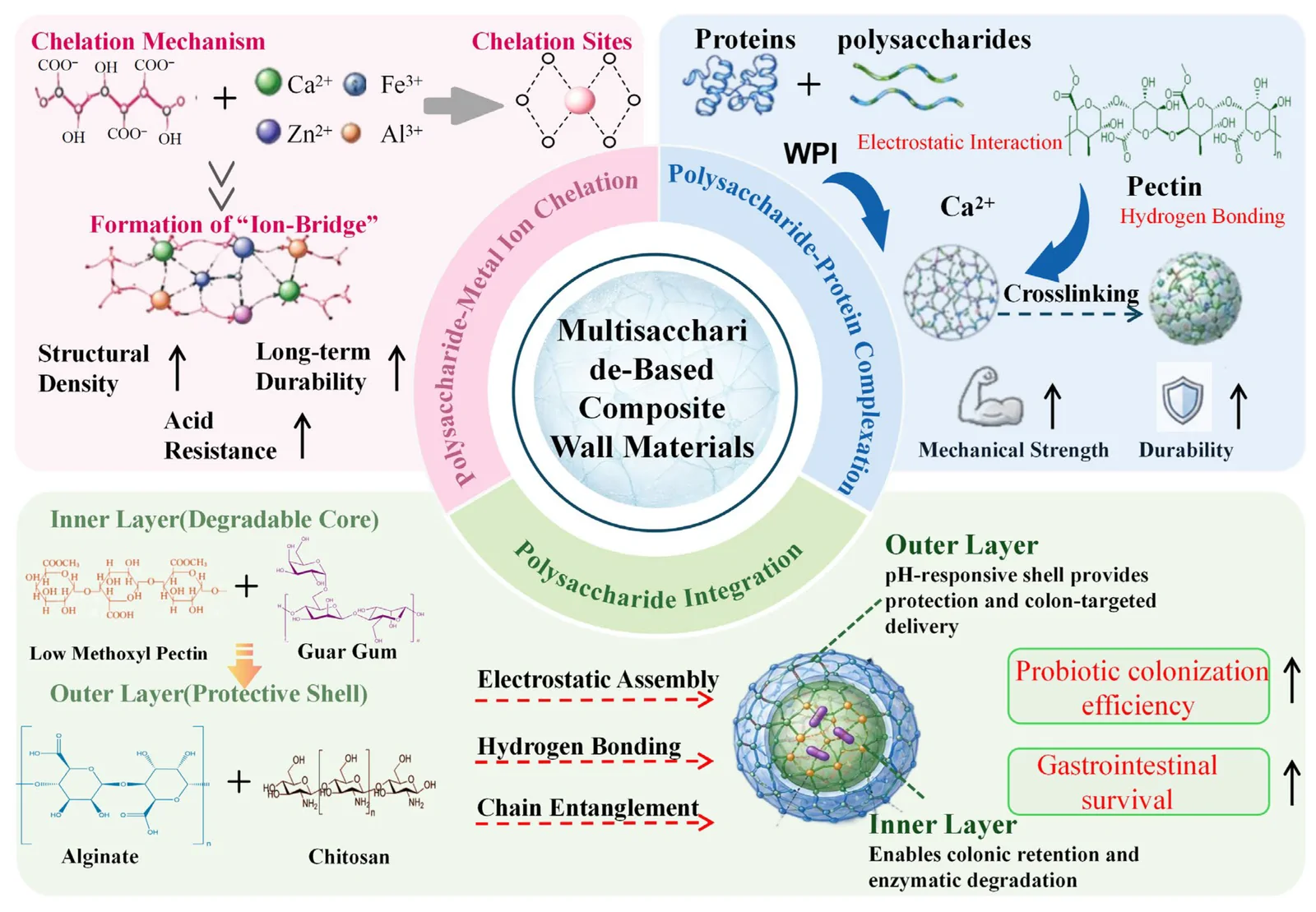
Polysaccharide-based composite wall materials assembly mechanisms driven by polysaccharide–polysaccharide interactions, polysaccharide–protein complexation, and polysaccharide–metal coordination, which enhance structural integrity, acid tolerance, and controlled release capacity.

Moreover, because of its film-forming properties, GG is ideal for use in edible films for food packaging applications [65]. González-Cuello et al. [61] found that microencapsulated *Limosilactobacillus reuteri* using binary mixtures of gellan gum and calcium via internal ionic gelation improved encapsulation efficiency and probiotic survival. While GG’s limited mechanical performance, comparatively high gelation temperature, and instability under physiological conditions hinder its practical application, chemical and physical modifications can help to overcome these limitations. In the first approach, gel strength and viscosity are controlled by adjusting environmental parameters such as temperature, pH, and shear force. In the second approach, crosslinking agents are added to strengthen the gel network and improve transparency [63]. Combining GG with other polymers could increase gel strength and enhance probiotic cell viability in stomach fluid simulations [66]. Najafpour et al. [67] developed composite microcapsules by adding gellan fluid gel to alginate hydrogel beads that contained *L. reuteri*. In addition to enhancing probiotic viability under simulated gastrointestinal and heat stress conditions, the resultant beads were characterized by improved hardness and diameter uniformity. The assembly mechanisms of polysaccharide-based composite wall materials, driven by polysaccharide–polysaccharide interactions, polysaccharide–protein complexation, and polysaccharide-metal coordination, are schematically shown in Figure 2.

### 2.4. Xanthan Gum

The Gram-negative bacterium *Xanthomonas campestris* has been used to ferment xanthan gum (XG), a branching heteropolysaccharide with a high molecular weight. Similarly to cellulose, the backbone has β-(1→4)-D-glucose residues; however, trisaccharide side chains are alternately linked between glucose units by α-(1→3) linkages [68]. XG has a pentasaccharide repeating unit with a molar ratio of 2:1:2 that contains D-glucose, D-glucuronic acid, and D-mannose. The anionic nature of the polymer along its side chains is attributed to glucuronic acid and pyruvate groups [69]. Through glucuronic acid residues attached to specific mannose units, XG exhibits distinctive branching architectures, giving this polysaccharide remarkable stability and functional activity [70]. Due to XG’s excellent solubility and biocompatibility, probiotics can be encapsulated without being harmed or degraded [71]. Additionally, XG can more effectively deliver probiotics due to its superior resistance to enzymatic destruction, gastrointestinal digestion, and heat and acid stability [72]. Relative to other biopolymers, XG has excellent film-forming ability and good mechanical strength. These characteristics offer effective protection for encapsulated probiotics against harsh environments, and its high viscosity at low concentrations makes it stable and allows for controlled release, thereby enhancing the overall probiotic delivery efficiency [73]. Shen et al. [74] used XG combined with κ-carrageenan to encapsulate *Lactobacillus acidophilus* JYLA-191 and experimentally confirmed that it not only improved the survival rate of probiotics after pasteurization but also enhanced their gastrointestinal activity, keeping them active in harsh environments. However, the application of XG alone is limited by its unpredictable hydration, unstable viscosity, and susceptibility to microbial contamination, while its mechanical strength further restricts its applicability in complex food systems [72,75]. Two approaches have been adopted to address these problems; namely, chemical modification and physical cross-linking. Chemical modification can be accomplished by cross-linking with citric acid as a cross-linker, resulting in XG blends with improved properties. Physical cross-linking is realized through ionic bonds, hydrogen bonds, and hydrophobic interactions, which enable XG to form polymeric networks with other polysaccharides or macromolecules [75], compensating for its limitations. Hou et al. [76] fabricated a composite gel based on *Bletilla striata* polysaccharide and XG via hydrogen bonding. This interaction increased XG’s viscosity and enhanced its structural stability.

### 2.5. Starch

One of the most common polymers in nature is starch. Starch is a biodegradable polymer with low production cost which is widely present in cereals, root and tuber crops, fruits, and plant leaves [77]. It mainly consists of two structural fractions: amylose and amylopectin [78]. Amylose is a linear polysaccharide composed of glucose units linked together via α-1,4 glycosidic linkages, with less branching and only one non-reducing end. In contrast, amylopectin is an extensively branched network with multiple non-reducing ends [79]. The excellent gelling ability and film-forming properties of natural starch make it an ideal wall material for microcapsules. When applied as a wall material, it forms a transparent shell over the core material, providing effective encapsulation [80]. Its rough surface offers anchoring points for probiotic cells, while the glycosidic backbone supports probiotic adhesion via interfacial interactions, significantly enhancing the efficiency of probiotic delivery [81]. Modified starch refers to a polysaccharide derived from chemical or physical native starch modification. It is often used for probiotic encapsulation, and it is among the most suitable materials for this purpose due to its multifunctional characteristics and ability to protect sensitive microorganisms [52]. It can also overcome some shortcomings of native starch. Modified starch can form a dense network structure that degrades slowly in the intestine, achieving the targeted release of probiotics and increasing their survival in simulated gastrointestinal fluid [53]. Furthermore, its porous structure, mechanical strength, and degradation characteristics can be modified through enzymatic treatment. In addition to ensuring probiotic stability during production, storage, and gastrointestinal transit, starch can also be used to develop effective probiotic carriers [17]. Liu et al. [82] encapsulated *Lactiplantibacillus plantarum* Heal 19 using β-cyclodextrin and resistant starch, and discovered that using these polysaccharides as wall materials greatly improved cell viability after storage and gastrointestinal digestion.

### 2.6. Cellulose

Cellobiose is the repeating disaccharide unit of cellulose, a naturally occurring linear polysaccharide composed of β-D-glucopyranose units connected by β-1,4-glycosidic linkages [83]. It possesses high mechanical strength and crystallinity. Through hydrophobic interactions and hydrogen bonds, these characteristics enable cellulose to form a dense protective network that serves as a physical barrier for probiotics [52,84]. Furthermore, cellulose’s insolubility in the digestive tract prevents gastric fluid infiltration during gastrointestinal transit and helps to preserve the mechanical integrity of encapsulation systems [84]. Cellulose exhibits exceptional film-forming performance, enabling the construction of protective barriers ensuring the controlled release of probiotics in the gastrointestinal tract, thereby allowing a large number of viable cells to reach the intestine and exert beneficial physiological effects [85]. Dietary fiber cellulose also supports digestive health, making it a useful addition to probiotic compositions.

Cellulose can be chemically modified to produce a variety of derivatives, including carboxymethyl cellulose and microcrystalline cellulose, for which its enhanced properties make them more suitable for probiotic encapsulation [86]. Zhu et al. [87]. created probiotic microcapsules based on gelatin/carboxymethyl cellulos, and showed that the probiotics had great vitality after digestion and that the microcapsules had excellent storage stability. Li et al. [88] fabricated a three-dimensional cellulose-based hydrogel network using *Millettia speciosa* Champ cellulose and carboxymethyl cellulose to encapsulate *Lacticaseibacillus paracasei* BY2. The system demonstrated satisfactory encapsulation efficiency and a sustained release profile. These studies emphasize the importance of cellulose-based polymers in encapsulating probiotics. Nanocellulose, for instance, is a multifunctional and renewable nanomaterial derived from cellulose [89]. Nanocellulose combines excellent mechanical strength, large specific surface area, biodegradability, and good biocompatibility. With these advantages, it can effectively resist harsh external environments and protect probiotics [89,90]. In probiotic nanoencapsulation, although food-grade polysaccharides alone offer favorable biocompatibility, they struggle to simultaneously achieve adequate mechanical properties and colon-targeting functionality. To overcome this limitation, current strategies primarily employ polysaccharide blends or polysaccharide–protein composite matrices. However, in some systems, synthetic polymers serve as the main carrier matrix, with polysaccharides used only as minor additives. This distinction is essential for accurately interpreting each delivery system’s design rationale [91]. The effects of structural and physicochemical properties of polysaccharides on probiotic encapsulation are summarized in Table 1.

## 3. Probiotic Encapsulation Technologies

Encapsulation technology forms a physical barrier to protect probiotics from damage in adverse environments. During encapsulation, the active components are encapsulated within polymer coatings, generating spherical particles to achieve probiotic protection and targeted intestinal delivery [92]. This section introduces a range of probiotic encapsulation techniques, covering extrusion, spray drying, freeze drying, electrospraying, electrospinning, layer-by-layer self-assembly, and other methods. Encapsulation efficiency and probiotic viability comparisons across studies are severely limited by methodological heterogeneity. Considerable variability exists among protocols for simulated gastrointestinal digestion, which can substantially affect measured survival rates [93]. Strain specificity plays a critical role in determining tolerance to acid, bile salts, and digestive enzymes, as distinct strains exhibit different levels of tolerance to these harsh conditions [94]. Methods used for viability assessment also vary considerably across studies. Alough plate counting remains the most widely used technique, it has inherent limitations—notably bacterial aggregation, which often causes underestimation of viable counts and compromises the comparability of data across trials [95]. Fluorescence microscopy and flow cytometry have also been applied for probiotic viability assessment [96], and the emergence of bioelectronic alternatives further underscores the methodological diversity in viability assessment [97]. Numerous factors can influence the analysis results, and a comprehensive perspective is essential. A more meaningful approach entails examining general trends within each study under consistent experimental conditions and interpreting the reported data in consideration of the experimental methodologies, rather than comparing absolute values across studies. To this end, relevant technologies and their advantages and disadvantages are summarized in Table 2.

### 3.1. Extrusion

**Figure 3:**
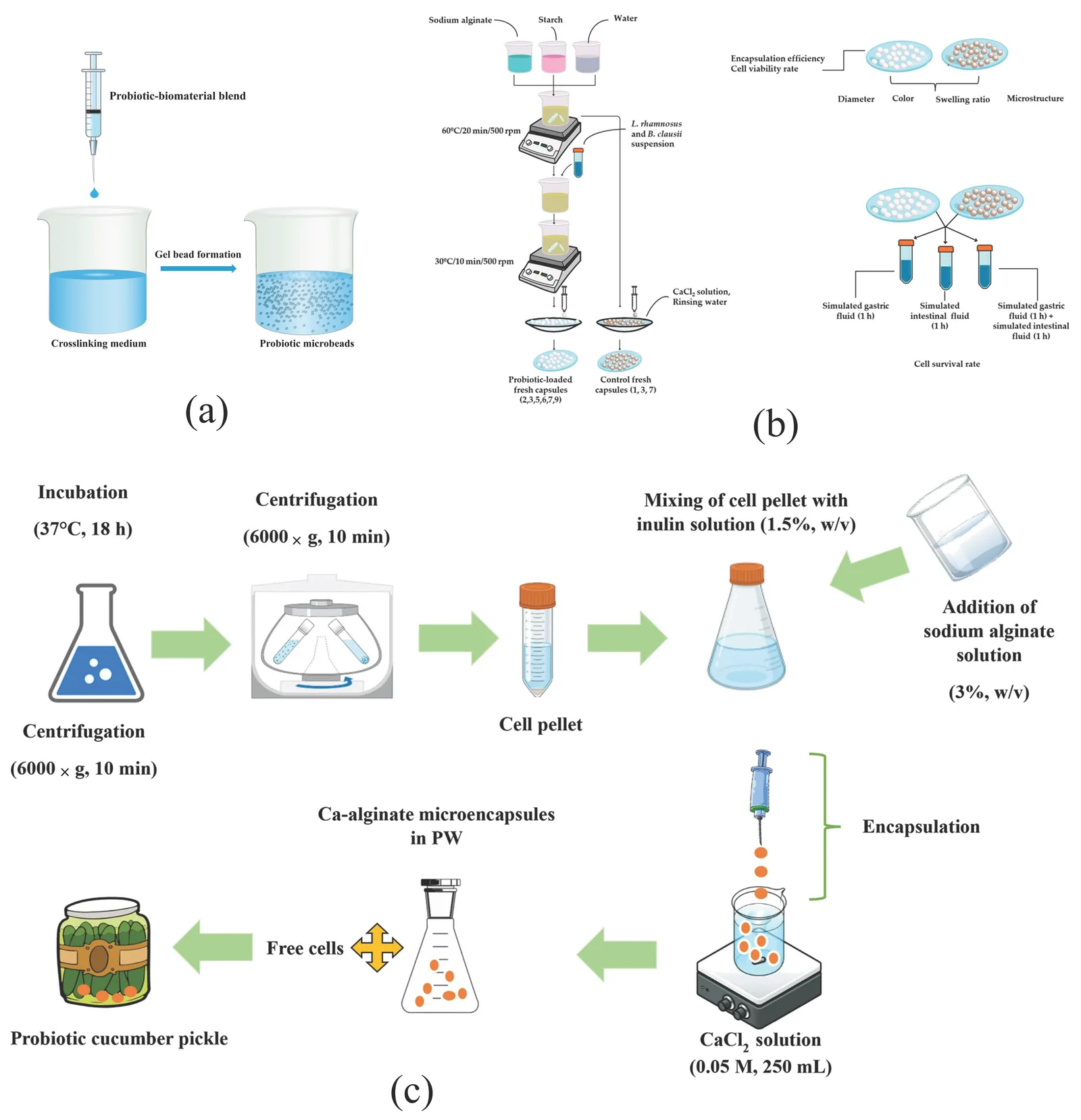
Depictions of extrusion processes. (**a**) Preparation of probiotic microcapsules using the extrusion technique. (**b**) Extrusion-based microencapsulation of *L. rhamnosus* and *B. clausii.* Reproduced from [119] under the terms of the Creative Commons CC BY 4.0 license. (**c**) Microcapsule fabrication using extrusion technology. Reproduced from [122] under the terms of the Creative Commons Attribution 4.0 International License.

One of the most effective encapsulating techniques is extrusion. Probiotics are kept active in the human stomach through extrusion, which provides a physical barrier. After combining the probiotic suspension with a polymeric solution, the combination is extruded into a coagulation bath, where the probiotics become trapped in the polymeric network that is formed [117]. Ullah et al. [118] used extrusion to encapsulate *Bifidobacterium longum* BL-101 and *L. acidophilus* LA-832. According to the test results, the encapsulated probiotic cultures showed good tolerance to simulated gastric juice and bile salts. Scanning electron microscopy (SEM) imaging revealed clear micropores dispersed throughout sodium alginate microbeads. Gheorghita et al. [119] used wheat starch and sodium alginate as wall materials to create microcapsules co-encapsulating *Bacillus clausii* and *L. rhamnosus*. These studies not only demonstrated that the fabricated microcapsules have a structurally regular network and uniform phase distribution but also proved that extrusion technology can improve the survival rate of probiotics, which is mainly due to the abundant hydroxyl groups in starch generating hydrogen bonds with sodium alginate’s carboxylate groups. Baleshzar et al. [120] optimized the extrusion process and encapsulated *Lacticaseibacillus rhamnosus* by employing a two-layer extrusion encapsulation technique. Alginate formed the inner layer of the microcapsules, while jujube mucilage combined with whey protein isolate made up the outer layer. When JM and WPI were blended at a ratio of 6:4, the encapsulation efficiency of microencapsulated *L. rhamnosus* reached 94.16%, and the probiotics’ survival rate after 15 min of heat stress treatment reached 69.14%. SEM revealed that the addition of microcapsules reduced the number of pores and voids, resulting in a denser microstructure within the product. This technique provides a novel approach for probiotic dairy product process optimization and quality improvement. Although the extrusion method is low-cost and effective, the microcapsules produced typically exceed 200 micrometer, whereas the microcapsule size should be below 100 μm to maintain a desirable food product texture. Although the encapsulation size does not directly impact efficiency, it affects the sustained release profile of bioactive substances and modulates the physicochemical attributes of the microcapsule matrices [121]. The extrusion techniques for probiotic encapsulation are summarized in Figure 3.

### 3.2. Spray Drying

**Figure 4:**
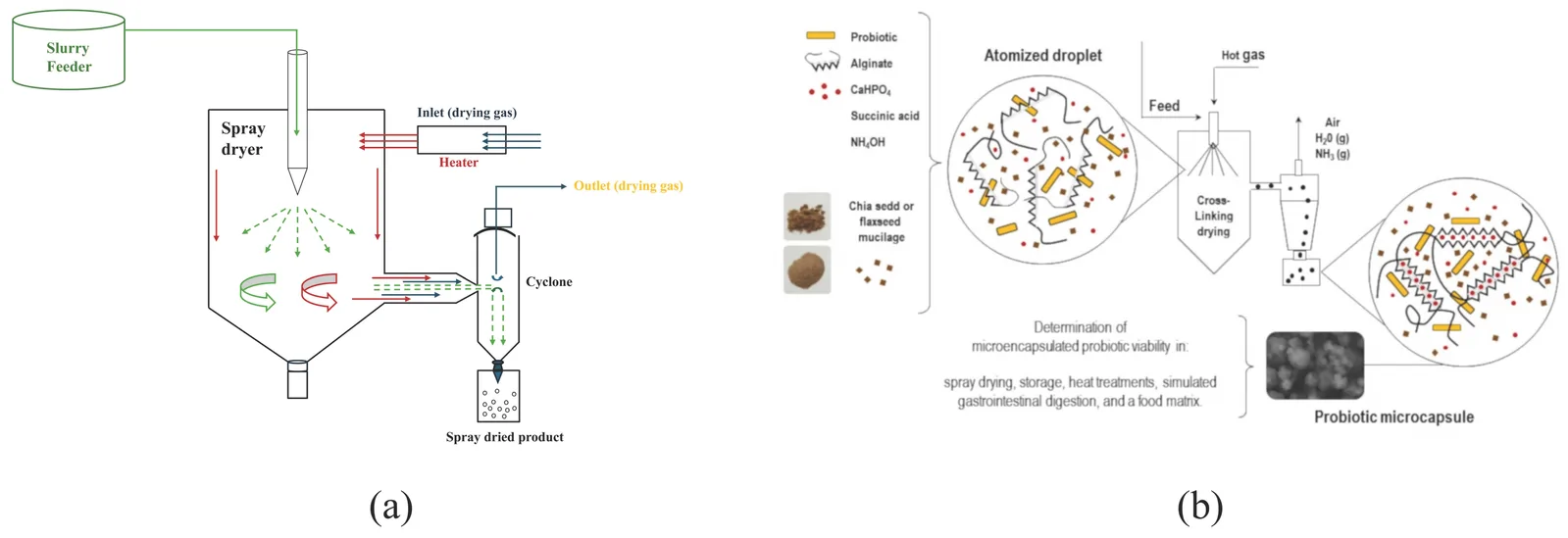
Depictions of spray drying processes. (**a**) General spray-drying technique. (**b**) Preparation of probiotics encapsulated in cross-linked alginate matrices incorporated with chia seed or flaxseed mucilage via spray drying. Reproduced from [129] under the terms of the Creative Commons CC BY 4.0 license.

Spray drying is a highly efficient microencapsulation approach. Its underlying mechanism involves the atomization of a polymeric solution or suspension containing probiotics into minute droplets, prior to swift evaporative drying in a heated airstream to generate microcapsules [52,123]. It also enables continuous processing at high throughput. Operational parameters including inlet air temperature, feed rate, and atomization pressure can be finely tuned such that the particle size distribution and encapsulation efficiency the of produced microcapsules are optimized [124,125]. Spray drying is usually adopted in industrial production due to advantages such as high preparation efficiency, low cost, and scalability for mass production [101]. D’Amico et al. [103] used a mixture of several matrices containing poly(methyl acrylate-co-methyl methacrylate-co-methacrylic acid), inulin, sodium alginate, and/or maltodextrin for spray drying of *B. longum* (strain 22348). Their experiment produced a yield of 90% and an encapsulation rate of 97%. Probiotics can be successfully protected during production, storage, and gastrointestinal simulations through the use of this technique. The procedure is more productive and uses less energy than freeze-drying methods, making it appropriate for large-scale industrial manufacturing. To further protect probiotics, some studies have further refined the wall material formulations. *L. rhamnosus* GG was encapsulated by Yin et al. [126] using spray drying and a water-in-oil-in-water double emulsion that included solid fat. The SEM and fluorescence microscopy images verified that probiotics were encapsulated within the double emulsion matrix, increasing survivability from 43.23% to 65.16% while maintaining intact subcellular structures. To alleviate the adverse impact of thermal damage, spray-drying techniques for probiotics usually include protective agents such as prebiotics, sugars, and protein [127]. Boontun et al. [128] compared the protective effects of different agents on *Bifidobacterium animalis* subsp. *lactis* KMP H9-01 during the spray-drying process, finding that 5% (*w*/*v*) trehalose or skim milk provided the maximum viable cell count at an inlet temperature of 160 °C and an outlet temperature of 80 °C. In addition, the samples exhibited satisfactory stability after six months of storage at ambient temperature and retained the greatest number of viable cells. The spray drying techniques for probiotic encapsulation are summarized in Figure 4.

### 3.3. Freeze Drying

Freeze drying has been broadly accepted as a highly efficient drying approach to retain probiotic cell activity, benefiting from moderate processing conditions. Freeze drying technology circumvents thermal damage to probiotics through direct sublimation of moisture under low-temperature vacuum conditions, thereby enhancing bacterial survival rates [130]. Microcapsules fabricated using this technology have a loose, porous architecture showing excellent rehydration properties, allowing for the quick release of probiotics in the intestinal environment [131]. Screening and optimizing protectants are crucial for preserving probiotic cells during the freeze-drying process. Wang et al. [132] systematically investigated the effects of adding trehalose on *L. plantarum* LIP-1 during freeze drying. The protective effect observed was largely ascribed to the activation of metabolic pathways involving L-glutamic acid and cysteine during trehalose metabolism. Trehalose also decreased the degree of membrane damage during the desiccation phase by controlling the cysteine metabolic pathway, which raised the intracellular glutathione concentration and improved antioxidant levels. However, the long drying cycle and high energy consumption of freeze-drying technology limit its industrial application. To overcome these limitations, Acosta-Piantini et al. [133] developed an innovative flash freeze drying (FFD) technology. They microencapsulated *L. acidophilus* LA5 cells with calcium alginate and chitosan and evaluated under three FFD temperature conditions. The total drying time was reduced from 2880 min using conventional freeze drying technology to 900 min, a decrease of 68.75%, while improving the survival rate of the microencapsulated *L. acidophilus* LA5. Comparing freeze drying with spray drying also provides valuable guidance for selecting an appropriate drying process. Fu et al. [134] found that *L.rhamnosus* GG’s survival rate after spray–freeze drying decreased by 17.48%, compared to spray drying. This was attributed to ice crystal formation in the freezing stage, which potentially induced mechanical damage in cell membranes, thus limiting large-scale industrial applications of this technology [135,136]. Traditional spray drying causes a significant decline in survival rate due to high-temperature thermal stress. However, freeze drying requires high energy consumption, long production cycles, and significant capital investment in equipment.

### 3.4. Electrospraying

**Figure 5:**
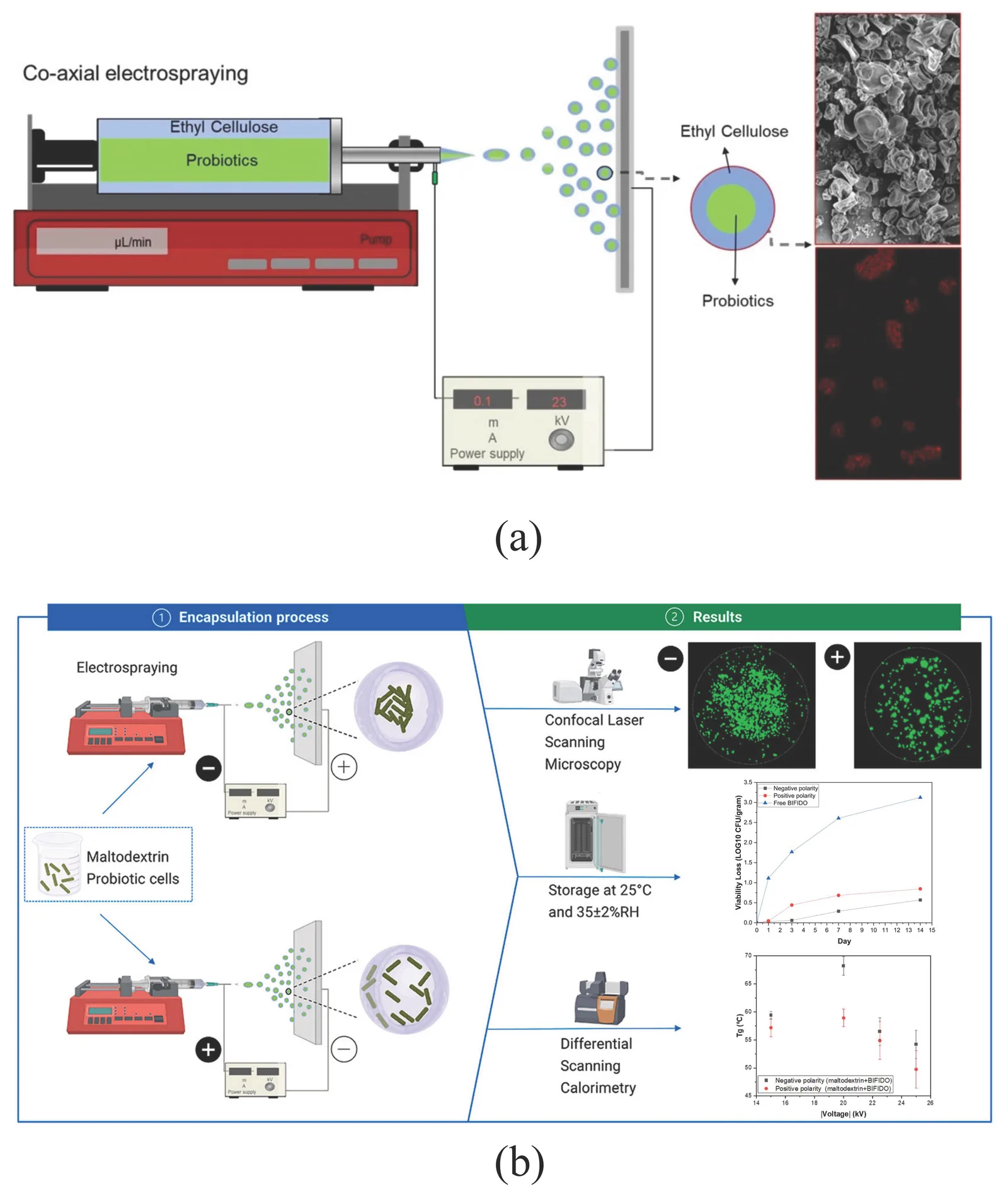
Depictions of electrospraying processes. (**a**)Co-axial electrospraying technique. Reproduced from [142] under the terms of the Creative Commons CC BY 4.0 license. (**b**) Encapsulation of *B. animalis* subsp. *lactis* using electrospraying and results of the study. Reproduced from [143] under the terms of the Creative Commons CC BY-NC-ND license.

Electrospraying technology is based on electrohydrodynamic principles, in which charged droplets are atomized under a high-voltage electric field and then dried to form microcapsules [137]. Due to its mild processing conditions, controllable particle distribution and size, and high encapsulation efficiency, electrospray technology has attracted extensive research interest for the delivery of probiotics [138]. To further investigate the potential application of electrospraying for probiotic encapsulation, Laina et al. [139] used inulin and whey protein isolates as composite matrices to encapsulate *L. rhamnosus* LGG via electrospraying and compared the electrospraying method’s efficiency with that of traditional freeze-drying techniques. Their results showed that the encapsulation efficiency of the electrospraying group was slightly lower than that of the freeze-drying group. However, the electrospraying treatment yielded a 93% survival rate for *Lactobacillus rhamnosus* LGG in harsh gastric environments, resulting in higher tolerance to gastric stress. Additionally, Namazifar et al. [140] established a pH-responsive encapsulation route targeting *L. plantarum* (ATCC 8014) using a peristaltic pump-assisted electrospraying approach. Two food-grade hydrogel matrices were adopted: a binary alginate-starch system and a novel ternary alginate-pectin-starch system. Both encapsulation systems significantly enhanced the probiotic’s survival rate after 2 h in simulated gastric fluid at pH 2. Ma et al. [141] used an electrospraying method to co-encapsulate the probiotic *L. plantarum* KLDS 1.0328 (LP KLDS 1.0328) and epigallocatechin gallate using whey protein concentrate as the base material. Their results demonstrated that the viable count of LP KLDS 1.0328 encapsulated in whey protein concentrate microcapsules remained stable at 6.82 log CFU/g following 28 days of storage at 4 °C, reflecting the strain’s enhanced storage stability. In comparison, the viable cell counts in free-floating cells decreased by 3.88 log CFU/g during the same period. However, the survival rate of encapsulated LP KLDS 1.0328 was lower than that of cells in the biopolymer solution. This phenomenon may be attributed to damage to probiotic cells caused by high pressure and osmotic stress during electrospraying. The spray drying techniques for probiotic encapsulation are summarized in Figure 5.

### 3.5. Electrospinning

**Figure 6:**
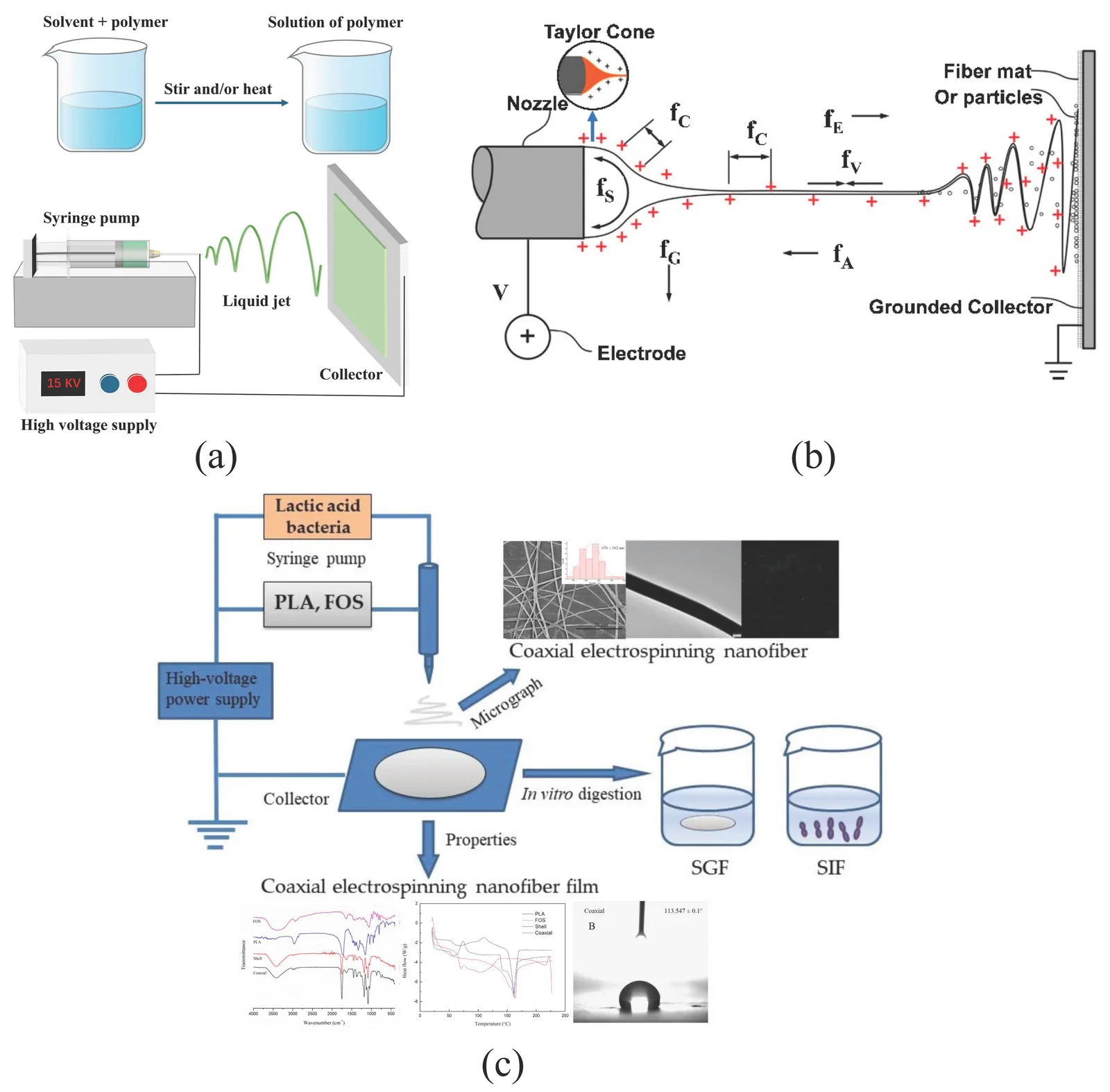
Depictions of eletrospining processes. (**a**) Single-needle electrospinning. (**b**) Jet evolution and force analysis during electrospinning. Reproduced from [151] under the terms of the Creative Commons CC BY 4.0 license. (**c**) Coaxial electrospinning. Reproduced from [152] under the terms of the Creative Commons CC BY 4.0 license.

Electrospinning technology utilizes a high-voltage electric field to draw polymer solutions into nanoscale fibers, forming three-dimensional porous scaffolds with high specific surface areas enabling the physical encapsulation and protection of probiotics [144]. Unlike microcapsules fabricated via electrospraying, electrospun fiber membranes have inherent porous structures and fiber microstructure which can be regulated. The enhancing effect of electrospinning on probiotics’ gastrointestinal tolerance have been verified through experimental studies [145,146]. Nawaz et al. [147] used a composite of polyvinyl alcohol, carboxymethyl cellulose, and sodium alginate as the matrix to encapsulate *L. rhamnosus* GG (ATCC 53103) via uniaxial electrospinning, reaching an encapsulation efficiency of 82.06%. SEM confirmed that the probiotics were effectively embedded within the fibers, and the nanofibers’ continuous, defect-free structure provided a physical barrier for the probiotic cells. In this formulation, the blend’s spinnability depended on polyvinyl alcohol, as only the formulation containing 50% PVA (R5) yielded electrospun nanofibers, whereas lower PVA ratios (R1–R4) were unspinnable. However, electrospinning is affected by factors including the voltage magnitude and polymer solution properties, in which can influence the survival of probiotics [148]. To address these disadvantages, coaxial electrospinning has been used to construct core–shell structures. Tan et al. [149] developed polyvinyl alcohol-fucoidan@ethyl cellulose (PVOH-FUC@EC) core–shell electrospun nanofibers via coaxial electrospinning to co-encapsulate *L. plantarum* 69-2 (LP69-2) and dihydromyricetin. Ultimately, embedding LP69-2 within PVOH-FUC@EC core–shell nanofibers significantly enhanced its survival rate during simulated gastrointestinal digestion. In this coaxial electrospinning system, ethyl cellulose served as the shell matrix, while the core was composed of polyvinyl alcohol into which fucoidan was introduced. Polyvinyl alcohol/fucoidan blend nanofibers have also been fabricated via electrospinning to co-encapsulate probiotics and polyphenols, which could elevate probiotics’ survival rates and boost antioxidant performance. However, it remains unclear how different polyphenol concentrations affect the viability of probiotics [150]. In summary, electrospinning can enhance the encapsulation rate and survival efficiency of probiotics, demonstrating promising application prospects. The spray drying techniques for probiotic encapsulation are summarized in Figure 6.

### 3.6. Layer-by-Layer Assembly

**Figure 7:**
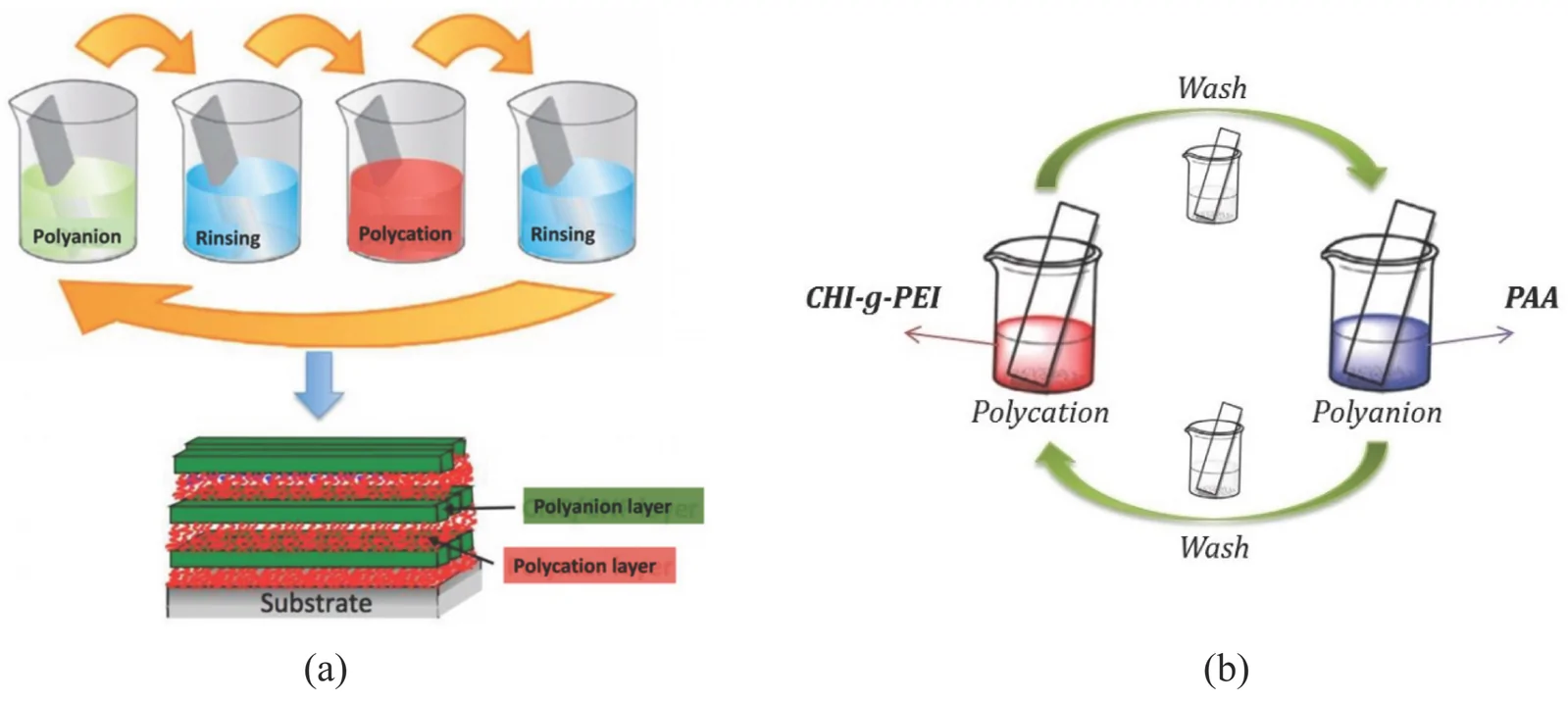
(**a**) Diagram of the LBL deposition process. Reproduced from [160] under the terms of the Creative Commons CC BY 4.0 license. (**b**) LBL film formation with polyethyleneimine-grafted chitosan and polyacrylic acid. Reproduced from [161] under the terms of the Creative Commons CC BY 4.0 license.

Layer-by-layer (LBL) assembly encapsulation draws on electrostatic interactions between polymer materials with opposite charges on the substrate surface to gradually deposit polymers and form a multilayered structure [153,154].Layer-by-layer assembly enables probiotics to resist harsh processing conditions and unfavorable gastrointestinal conditions. Li et al. [155] used this technology to encapsulate *L. plantarum* 90, thus achieving a survival rate of over 60% under harsh acid, bile salts, and simulated gastrointestinal conditions. Liu et al. [156] used the anti-solvent method and electrostatic layer-by-layer assembly to co-encapsulate polyphenols and probiotics, thereby improving the probiotics’ antioxidant activity and storage stability. Furthermore, the number of encapsulation layers significantly influences probiotic stability. After 4 weeks of refrigeration, probiotics encapsulated in four layers retained a viable cell concentration of 10.72 log CFU/g, which exceeded the 8.23 log CFU/g of free probiotics. Qian et al. [157] created an effective surface coating technique using tannic acid (TA) –Mg^2+^ complexes and casein phosphopeptide complexes to preserve probiotics and further enhance the effectiveness of layer-by-layer assembly. After being exposed to simulated digestive fluids, the encapsulated *Saccharomyces boulardii* (CNCM I-745) demonstrated good antioxidant performance, acceptable stability, and sustained viable cell count. Similarly, Wang et al. [158] used inulin and tannic acid/Ca^2+^ metal-phenolic networks (MPNs) as wall materials to enclose the anaerobic *Bifidobacterium lactis* through layer-by-layer assembly. This strategy strengthened the probiotics’ resistance against harsh gastrointestinal environments and improved their intestinal colonization capacity. The fabricated three-dimensional gel shell shielded encapsulated MPNs from degradation by gastric juice, boosting the tolerance of *B. lactis* to gastric acid and bile salts and raising the survival rate of the probiotics. In summary, as a preparation technique characterized by simple operation and mild conditions, layer-by-layer self-assembly can effectively overcome the poor inherent stability of single-layer wall systems by sequentially embedding various wall components [159]. The spray drying techniques for probiotic encapsulation are summarized in Figure 7.

### 3.7. Others

In recent years, growing interest in polysaccharide encapsulation technology for probiotics has boosted research in this field. Emulsification is a fabrication technique in which immiscible liquid phases are stabilized via a cross-linking agent to form an emulsion, following which the dispersed droplets are solidified. Subsequent to elimination of the oil phase and surfactants, stable microgel particles are obtained [63]. Peng et al. [162] utilized sodium alginate and soy protein isolate as wall materials to encapsulate *L. paracasei* ProSci-92 via the endogenous emulsification method. Under the optimized conditions of sodium alginate concentration at 20 mL/L, the encapsulation efficiency of the endogenous emulsification method reached 92.17%, which was higher than that of the extrusion method. Three-dimensional bioprinting technology employs bioinks comprising polysaccharide-based hydrogel matrices, bioactive agents, and other functional molecules as printing materials to fabricates precise 3D structures via layer-by-layer deposition [163]. Four probiotic strains (*Bifidobacterium bifidum*, *Bacteroides fragilis*, *L. rhamnosus*, and *Streptococcus thermophilus*) were co-encapsulated in alginate hydrogel filaments to create structured cocultures with multiple strains through a novel continuous chaotic bioprinting technique developed by Flores-Loera et al. [164]. This system was characterized by better probiotic viability than its monoculture equivalents, according to the reported experimental results. Effective cell counts were higher than 10^7^ CFU for at least six weeks under both ambient and refrigerated storage conditions when compared to unencapsulated probiotics. Microfluidic technology has also emerged as a promising strategy for the fabrication of polysaccharide-based microgels. When two immiscible fluid phases are introduced into microscale channels, uniform and stable droplets, particles, and hydrogel microspheres can be produced under precisely controlled shear forces [165]. Yazdani et al. [166] employed a microfluidic platform integrated with an aqueous two-phase system to engineer liquid-core hydrogel-shell microcapsules, enabling colon-targeted delivery of *Escherichia coli* Nissle 1917. The survival rate of probiotics during simulated gastrointestinal transit in the chitosan-coated formulation was 89%, while that associated with the Eudragit-chitosan-coated formulation was 95.2%. In this multilayer system, the hydrogel shell consisted of dextran and alginate. Chitosan was deposited as a polyelectrolyte layer, and Eudragit S100 formed an outer nanoparticle coating for enteric protection. In conclusion, many methods have been developed to encapsulate probiotics, and these approaches vary in their abilities to maintain cell viability, control particle uniformity, and accomplish targeted delivery. A comparative summary of biopolymer encapsulation, encapsulated probiotics, techniques, and improvements is provided in Table 3.

## 4. Applications in Dairy Products

### 4.1. Cheese

Cheese is a dairy product composed mainly of casein, milk fat, and other components from milk, with added yeast, salt, dyes, and other additives as permitted by regulations. Its production process mainly consists of the following three steps: acidification, coagulation, and dehydration [167]. Adding probiotics to processed cheese not only gives the product unique functional characteristics but also enhance its health benefits [168]. Cheeses’ low acidity, high pH, strong buffering capacity, and firm texture might offer effective probiotic protection during storage and gastrointestinal transit, allowing it to be adapted for probiotic encapsulation [169]. However, the dense structure of cheese may also limit the release of encapsulated probiotics [170]. Saeed et al. [171] compared *L. acidophilus* and *Lacticaseibacillus casei* encapsulated in sodium alginate–carrageenan microspheres with free cells. This encapsulation system effectively improved probiotics’ storage period of and mimicked gastrointestinal activity. After adding the microspheres into cheese, the cheese’s ash and protein contents and acidity improved, providing good sensory characteristics and improving its nutritional quality, to a certain extent. The addition of sodium alginate–chitosan microcapsules containing *L. acidophilus* La-05 to goat Ricotta cheese also improved its texture by reducing gumminess and adhesiveness. In addition, the encapsulated matrix in the microcapsule can limit the release of excessive organic acids and volatile fatty acids produced through probiotic metabolism, thereby improving the cheese’s overall flavor profile [172]. When using polysaccharide wall materials to encapsulate probiotics for cheese production, it is necessary to consider whether the inherent flavor of these materials affects the finished cheese’s sensory characteristics of. For example, chitosan and cupuacu powder, which have a slightly bitter and fibrous texture, can mask the natural dairy aroma of the cheese itself when added excessively [173]. When selecting different polysaccharide materials, it is therefore necessary to consider their applicability to various types of cheese, as well as addition ratios, and other factors.

### 4.2. Yogurt

Yogurt is a dairy product fermented from milk using strains such as *Streptococcus thermophilus* and *Lactobacillus delbrueckii* subsp. *Bulgaricus*. It has a unique sour taste [174]. The incorporation of probiotics into yogurt can effectively suppress the proliferation of pathogenic bacteria and regulate the microbiota in the intestinal tract, thereby promoting digestion and absorption and enhancing the body’s immunity [175]. Yogurt’s low pH and high moisture content pose challenges for ensuring the viability of probiotics, requiring encapsulation systems with sufficient acid resistance to maintain structural integrity [176,177]. Encapsulation is an effective method for improving probiotic viability. Kakili et al. [178] chose alginate and carboxymethyl cellulose as composite wall materials. The probiotic strain *Limosilactobacillus fermentum*, obtained from Malagheh yogurt, was encapsulated through extrusion and added to yogurt. The encapsulated probiotic cells had significantly greater viability than free cells (*p* < 0.05). Selecting suitable wall materials for probiotic encapsulation can effectively ensure the probiotics’ viability and improve the quality of yogurt. Milk with microcapsules of alginate–inulin containing *L. fermentum* 4-17 for yogurt manufacturing was developed by Ebrahimi et al. [179], which can improve product consistency, water-binding ability, and gel structure stability. The addition of inulin at 1.0% and 1.5% enhanced encapsulation efficiency and improved probiotic survival to over 75%. In recent years, innovations have been introduced based on traditional probiotic encapsulation technology. For instance, *L. plantarum* Y42 has been encapsulated with sodium-alginate and chitosan, and microcapsules were further incubated to form biofilm microcapsules. The bacteria formed a biofilm structure by constructing a biofilm-microcapsule composite system, which significantly improved the strain’s tolerance to adverse environments and improved the quality and nutritional value of the product (*p* < 0.05) [180]. However, actual production still faces numerous challenges. When added in excess, high-viscosity polysaccharides such as chitosan and sodium alginate can cause aberrant gelation. They can also make yogurt excessively viscous, leading to problems like whey separation [175]. Therefore, when selecting polysaccharide materials, factors such as addition ratio and application should be considered [181].

### 4.3. Ice Cream

Ice cream is a frozen dairy product with a creamy, semi-solid consistency. It can be used to deliver probiotics as its pH level and total solids create the perfect conditions for bacterial viability [182]. Probiotic strains added to ice cream can improve intestinal digestion and nutrient absorption, preventing the growth of some harmful bacteria and enhancing host immune responses [168]. Given that probiotics are susceptible to a variety of unfavorable conditions, maintaining their survival rate is of utmost importance [183]. Encapsulation technology plays a crucial role in enhancing probiotic viability. Ice crystal formation and solute concentration buildup during ice-cream processing can induce mechanical and osmotic damage to probiotics [184]. To demonstrate that a microcapsule system may successfully lower bacterial loss, Farias et al. [185] encapsulated *L. rhamnosus* and *L. casei* using a calcium alginate–chitosan extrusion technique and added them to yellow myrtle berry ice cream. There has been increasing interest in the use of polysaccharide microcapsules to improve the physicochemical properties of food products. Using a combination of 0.6% (*w*/*w*) *ora-pro-nobis* mucilage and 0.9% (*w*/*w*) sodium alginate, de Morais et al. [182] created microcapsules containing *L. acidophilus* ATCC 4356 via ionic gelation. The beads were coated with either 5.25% (*w*/*w*) whey protein concentrate or 1.2% (*w*/*w*) chitosan before being added to ice cream. The encapsulated probiotic was found to greatly improve storage stability, with chitosan-coated microparticles showing the highest encapsulation efficiency (98.28%). It also positively influenced the final product’s viscosity and melting behavior, without impacting overrun, firmness, pH, or titratable acidity. Microcapsules made with guar or xanthan gum also have better cohesion, uniformity, and structural stability. They allow for more even distribution throughout the frozen dessert matrix and strengthen molecular associations. Consequently, the finished product’s textural and organoleptic characteristics of were notably improved [186]. The selection of appropriate wall materials is key to controlling the quality of ice cream. In practice, the polysaccharide concentrations should be carefully controlled, as excessive polysaccharides will significantly increase the mixture’s viscosity. Moreover, excess polysaccharide molecules form dense colloidal networks that prevent air incorporation and reduce overrun during freezing, while also causing a hard, granular texture and a sticky and rough mouthfeel [184].

### 4.4. Non-Fermented Dairy Beverages

Consumer demand for highly nutritious, functional health beverages continues to grow, driving major changes in the global beverage market. Probiotics, bioactive compounds, and other additives have been added to beverages as a result of this trend. Maintaining these components’ stability and bioactivity throughout production, refigeration, and passage through the human digestive tract remains a significant industrial challenge [187]. A composite emulsion was created using pectin and whey protein isolate to encapsulate *Bifidobacteria* in a dairy beverage system. This carrier formed a gel network that allowed for targeted delivery to the colon (*p* < 0.05) and effectively protected *Bifidobacteria* throughout simulated gastrointestinal transit [188]. In addition to emulsions, microencapsulation is one of the most widely employed strategies for protecting probiotics and enabling targeted delivery. Athayde et al. [189] used extrusion–emulsion to create chitosan–sodium alginate capsules containing *L. rhamnosus* GG, which they then added to ultra-high-temperature (UHT) dairy products. The extrusion and emulsification techniques achieved high encapsulation efficiencies of 86.01% and 74.43%, respectively, and the encapsulated probiotics maintained high survival rates (>89%) in UHT and powdered milk during storage. Without significantly altering the final product’s color, flavor, or texture, the encapsulated probiotics demonstrated enhanced viability, thermostability, gastric acid resistance, and salt tolerance. In contrast, spray drying and extrusion result in microcapsules with comparatively large particle sizes. These particles are visible in dairy products and cause undesirable sensory defects such as uneven texture distribution and gritty mouthfeel. As promising candidates for integration into various food matrices, nanocapsules have garnered considerable attention [190].

### 4.5. Others

**Figure 8:**
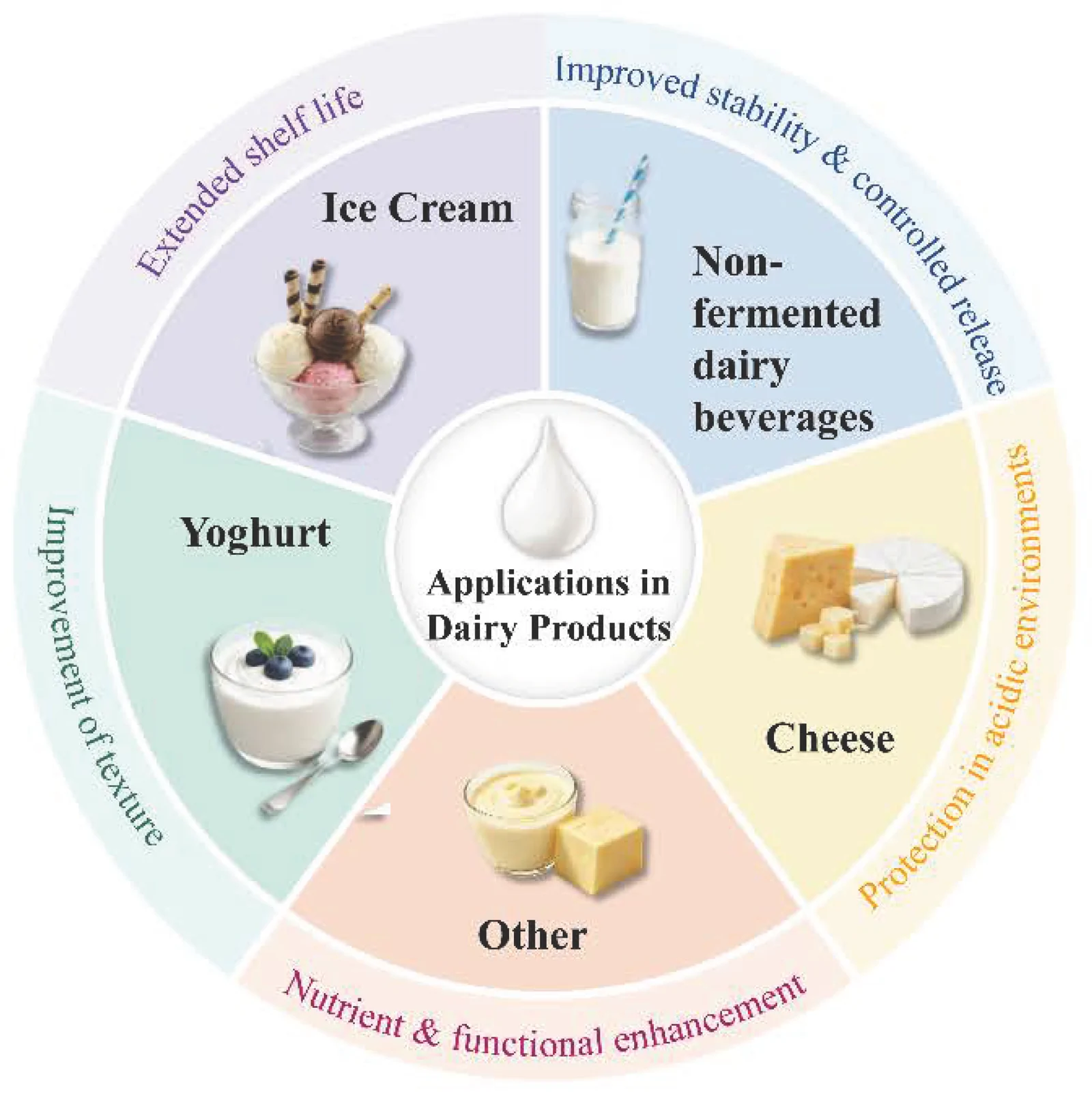
Schematic diagram of polysaccharide-based probiotics applied in dairy products.

Dairy products are excellent carriers for probiotic encapsulation because they are widely consumed and well-liked by consumers. Besides yogurt and cheese, several studies have applied polysaccharide-based encapsulation to other dairy substrates, such as butter and condensed milk [191]. Butter, cream, and sweetened condensed milk are examples of milk-fat-based products that have high fat contents and are primarily characterized by their milk fat fraction. While probiotic survival is hampered by high fat content and low water activity during production, storage, and gastrointestinal transit, polysaccharide encapsulation offers a viable approach to overcoming these challenges [192]. To encapsulate *L. acidophilus* and *B. animalis* ssp. *lactis*, Kaushik et al. [193] used whey protein hydrolysate–maltodextrin. Encapsulation enhanced probiotic viability and survival in butter during storage under both frozen and refrigerated conditions. Silva et al. [194] investigated how encapsulation affected butter quality. The researchers used extrusion-assisted ionotropic gelation to encapsulate *L. acidophilus* in sodium alginate; the resulting microcapsules not only retained higher probiotic viability in butter than free cells but were also characterized by enhanced hardness and functional properties. High osmotic pressure from sweetened condensed milk reduces shelf stability and hinders probiotic survival. Probiotics that are encapsulated in casein–sodium hyaluronate before being added to condensed milk decreased osmotic damage, extended shelf life, and altered microbial metabolism during fermentation. The condensed milk matrix’s flavor profile and general quality were ultimately enhanced by these protective effects [195]. For products such as butter or sweetened condensed milk, various wall materials and encapsulation techniques can be customized to enhance probiotic viability and product quality. The application of polysaccharide-based probiotics in dairy products is summarized (Figure 8).

## 5. Conclusions and Perspectives

This review elaborated on the structural properties of polysaccharides and their performance in encapsulating probiotics. In addition, we summarized major polysaccharide-based strategies for probiotic encapsulation, as well as practical applications of polysaccharide-based microencapsulation approaches for probiotics in dairy matrices. This review suggests that applying probiotics encapsulated with polysaccharides may help in further developing current encapsulation techniques and producing cutting-edge functional dairy products. Encapsulation using polysaccharides has attracted increasing attention given its effectiveness in maintaining probiotic viability during processing, storage, and gastrointestinal transit. Polysaccharide wall materials have been found to enhance probiotic viability in dairy matrices against environmental challenges including acidic conditions, high temperatures, oxygen exposure, and mechanical shear. These encapsulation techniques may contribute to improvements in gut microbiota modulation, antioxidant capacity, and antibacterial activity, in addition to increasing cell viability. As such, they may be used to expand the functional properties of probiotic dairy products. However, several bottlenecks still need to be addressed when applying this technology in industrial production contexts. First, the inconsistent particle size of microcapsules and poor reproducibility in large-scale production have not been resolved. Moreover, the complicated multi-layer encapsulation process involves multi-step crosslinking reactions, which greatly increases production cost and technical barriers. Second, the currently available polysaccharide wall materials have certain shortcomings, including those relating yo their mechanical strength, oxygen barrier properties, and targeted release characteristics. Realizing controlled probiotic release in different segments of the gastrointestinal tract is a critical scientific challenge that urgently needs to be addressed. Furthermore, adding encapsulated particles might affect the texture and flavor of dairy products, and the odors or rough textures induced by some polysaccharide wall materials can reduce the commercial competitiveness of the end products. The reviewed encapsulation systems vary considerably in their suitability for food applications. Wall materials such as alginate and pectin are food-grade polymers with excellent safety and widespread utilization in commercial food products. In contrast, most systems based on synthetic polymers or non-conventional cross-linkers remain confined to laboratory-scale investigations and have not yet been validated for food use, for instance, glutaraldehyde—a common synthetic cross-linker used in laboratory-scale studies, is known to be cytotoxic and, thus, unsuitable for food applications. As such, a clear distinction should be made between formulations that are ready for food applications and those that require further safety testing, including cytotoxicity and toxicological studies. To enable industrial-scale production, it will be important to develop automated and continuous encapsulation platforms. Streamlining cross-linking procedures and optimizing processing parameters could improve batch-to-batch consistency and reduce manufacturing costs, thereby boosting production efficiency and reproducibility. An intriguing direction for next-generation probiotic delivery systems includes intelligent wall materials that respond to environmental stimuli such as pH, digestive enzymes, and metabolites produced by the gut microbiota. Compared to traditional encapsulation matrices, these materials have enhanced targeting performance, allowing for site-specific probiotic release, especially in the colon. Another challenge is maintaining favorable sensory properties without sacrificing encapsulation efficiency. Future studies may modify the molecular structure of wall materials alongside flavor-masking strategies to improve the taste and overall acceptability of probiotic products while meeting consumers’ expectations for clean-label formulations. From a regulatory perspective, establishing standard protocols for the toxicological assessment and performance evaluation of encapsulation wall materials will be crucial. Greater international regulatory standard harmonization can also be expected to facilitate the approval and commercialization of novel encapsulation materials, laying a solid foundation for industrial translation. Ultimately, future research should establish quantitative models correlating alginate M/G ratios with hydrogel network architectures and probiotic release kinetics, as the M/G ratios determine gel strength, swelling behavior, and gastrointestinal release profiles. To facilitate evaluation, future studies should systematically report the experimental parameters used in encapsulation efficiency determination, such as bacterial enumeration methods.

## Figures and Tables

**Table 1:** Effects of structural and physicochemical properties of polysaccharides on probiotic encapsulation.

Types of Polysaccharides	Structural Characteristics of Different Polysaccharides	Charge Properties	Protective Effects for Probiotics	Core Physical and Chemical Advantages and Applications	Main Research Conclusions	Refs.
Sodium alginate	Natural linear anionic polysaccharide mainly composed of β-D-mannuronic acid (M) and α-L-guluronic acid (G) linked by (1→4) glycosidic bonds. The M/G ratio determines the gel structure, porosity, and mechanical strength. High-G alginate forms stronger and denser gels with improved integrity.	Anionic polysaccharide containing carboxyl groups (-COOH/-COO^−^). Calcium ions interact with guluronic acid residues to form ionic crosslinked networks based on the “egg-box” model.	Calcium alginate hydrogels provide protection against gastric acid and bile salts. The pH-responsive swelling behavior allows for compact structures in acidic environments and controlled release in intestinal conditions.	Excellent biocompatibility, biodegradability, mild gelation conditions, and oxygen barrier properties. Widely applied in probiotic beads, hydrogel systems and microencapsulation delivery platforms.	Although SA is a widely used probiotic wall material; its high porosity, weak mechanical strength, and insufficient stability limit its independent use. Combination with other polymers improves encapsulation efficiency and probiotic survival.	[32,33,34,35,36,38,41,42]
Pectin	Plant-derived heteropolysaccharide mainly composed of D-galacturonic acid units linked by α-(1→4) glycosidic bonds. According to the degree of esterification (DE), pectin is classified into low-methoxyl pectin (LMP) and high-methoxyl pectin (HMP).	Anionic polysaccharide with free carboxyl groups. Low-methoxyl pectin forms calcium-mediated ionic gels through the “egg-box” mechanism, whereas high-methoxyl pectin forms gels mainly through hydrogen bonding and hydrophobic interactions.	Pectin hydrogels form protective barriers around probiotics, improving resistance against gastrointestinal stress and enhancing targeted intestinal delivery.	Excellent gel-forming ability, film-forming properties, biodegradability and compatibility with other polymers. Suitable for composite encapsulation systems.	Pectin improves encapsulation efficiency and mechanical stability when combined with sodium alginate, proteins, or other polymers. Composite systems further enhance probiotic viability and storage stability.	[47,48,49,50,52]
Gellan gum	Microbial extracellular polysaccharide produced by *Sphingomonas* species. The backbone consists of repeating tetrasaccharide units containing glucose, glucuronic acid, and rhamnose. GG undergoes coil-to-double helix transition during gel formation.	Anionic polysaccharide containing carboxyl groups. Divalent cations promote double-helix aggregation and intermolecular crosslinking.	GG-based gels provide physical protection against heat, acid, and enzymatic degradation. The three-dimensional network structure improves probiotic retention and controlled release.	Excellent gelation ability, thermal stability, flexibility, film-forming capacity and texture modification properties. Used in edible films, hydrogel beads, and probiotic delivery systems.	GG is a promising encapsulation material owing to its strong gel-forming ability. Its high gelation temperature and limited mechanical properties necessitate modification or combination with other polymers.	[59,60,61,62,63]
Xanthan gum	High-molecular-weight branched heteropolysaccharide produced by *Xanthomonas campestris*. The structure contains cellulose-like β-D-glucose backbone with trisaccharide side chains containing mannose and glucuronic acid residues.	Anionic polysaccharide due to glucuronic acid and pyruvate groups in side chains.	High-viscosity and stable network structure protect probiotics from environmental stress, gastrointestinal digestion, and enzymatic degradation.	Excellent rheological properties, high viscosity at low concentrations, acid resistance, thermal stability, and film-forming ability. Widely used as a stabilizer and encapsulation matrix.	XG provides effective probiotic protection and controlled release; its poor solubility and insufficient mechanical strength call for chemical modification or blending with other polymers.	[68,69,71,72,73,75]
Starch	Natural non-ionic polysaccharide mainly composed of amylose and amylopectin. Amylose is a linear α-(1→4)-linked glucan, while amylopectin contains α-(1→6)-linked branching points.	Generally non-ionic polysaccharide without charged functional groups. Modified starch derivatives may introduce functional groups to improve performance.	Starch-based coatings and networks protect probiotics during processing, storage and gastrointestinal transportation. Surface structure facilitates probiotic adhesion.	Abundant, low-cost, biodegradable, and safe food-grade material. Good gelation and film-forming properties. Modified starch improves stability and controlled release.	Native starch has limited functionality, but physical or chemical modification significantly enhances gel strength, degradation behavior, and probiotic protection efficiency.	[53,78,79,80,81]
Cellulose	Linear high-molecular-weight polysaccharide composed of β-D-glucopyranose units connected by β-(1→4)-glycosidic bonds. High crystallinity provides strong mechanical properties.	Mainly non-ionic polysaccharide. Cellulose derivatives such as carboxymethyl cellulose (CMC) introduce charged groups.	Cellulose creates physical barriers that protect probiotics from external stress and improve controlled release during gastrointestinal transit.	Excellent mechanical strength, film-forming ability, biodegradability, and functionalization potential. Nanocellulose provides high surface area and enhanced mechanical performance.	Cellulose derivatives and nanocellulose broaden probiotic encapsulation applications; native cellulose has limited hydrogel-forming ability and often requires chemical modification or combination with other polymers.	[83,84,85,89,90,91]

**Table 2:** Comparative evaluation of probiotic encapsulation methods.

Encapsulation Technology	Advantages	Disadvantages	References
Extrusion	Mild conditions. Low cost. High viability of the encapsulated cells.	Difficult for large-scale application. Difficulty obtaining small particles and dried products.	[98,99,100]
Spray drying	High preparation efficiency. Controllable scale. Continuous production.	Probiotic damage caused by high temperature and dry conditions. Poor microsphere size uniformity.	[101,102,103]
Freeze drying	Better stability of heat-sensitive substances.	Ice-crystal-induced damage to probiotic cells. Requires addition of cryoprotectants.	[104,105]
Electrospraying	Simple and cost-effective. Does not require additional reaction solvents.	Low yield. Not conducive to industrial-scale production.	[106,107]
Electrospinning	Enhanced probiotic stability and functionality. Processes can be carried out at room temperature.	High-voltage adverse impact on probiotics. Low yield.	[4,108]
Layer-by-layer assembly	Decreases microcapsule porosity. Improves microcapsule stability.	Low production efficiency. Increased microcapsule volume. Complex fabrication.	[109,110,111]
Emulsification	High probiotic survival rate. Achieve large-scale production of small-sized particles.	Adds fat as the dispersing phase. Irregular shapes and uneven size distribution.	[112,113]
Microfluidics	Microgels with uniform particle size and controllable shape. High embedding efficiency of active components.	Complex equipment. High operation costs.	[114]
3D printing	Prints complex porous geometric structures. Design flexibility.	Difficult to achieve mass production. Process parameters must be set precisely.	[115,116]

**Table 3:** Comparative summary of biopolymer encapsulation, encapsulated probiotics, encapsulation techniques, and improvements or advantages.

Biopolymers	Encapsulated Probiotics	Encapsulation Technologies	Encapsulation Efficiency (%)	Improvements/Advantages	Ref.
Sodium alginate, chitosan	*Lactobacillus acidophillus* LA-832, *Bifidobacterium longum* BL-101	Extrusion	93.60 ± 1.21	Remarkably enhanced tolerance towards bile salts and gastric fluid	[118]
Sodium alginate, wheat starch	*Lacticaseibacillus rhamnosus*, *Bacillus clausii*	Extrusion	96.7	Compact, highly ordered network matrices with homogeneous phase dispersion	[119]
Alginate, jujube mucilage, whey protein isolate	*Lacticaseibacillus rhamnosus*	Extrusion	94.16 ± 7.72	Achieved 94.16% encapsulation efficiency and 69.14% probiotic viability after 15 min thermal treatment	[120]
Poly (methyl acrylate-co-methyl methacrylate-co-methacrylic acid, Eudraguar biotic), inulin, sodium alginate, maltodextrin	*Bifidobacterium longum* (strain 22348)	Spray drying	97.1 ± 3.3	Obtained 97% encapsulation efficiency alongside a 90% production yield	[103]
Whey protein isolates	*Lacticaseibacillus rhamnosus* GG	Spray drying	NR	Increased the survival rate of probiotics from 43.23% to 65.16%	[126]
Trehalose	*Lactiplantibacillus plantarum* LIP-1	Freeze drying	NR	Alleviated cell membrane impairment during drying treatment	[132]
Calcium alginate, chitosan	*Lactobacillus acidophilus* LA5	Flash freeze drying	NR	Reduced the total drying time from 2880 min to 900 min by 68.75%	[133]
Inulin, whey protein isolate	*Lacticaseibacillus rhamnosus* GG	Electrospraying	82.67 ± 1.31	Achieved a survival rate as high as 93% under harsh gastric conditions	[139]
Alginate, starch, pectin	*Lactiplantibacillus plantarum* (ATCC 8014)	Electrospraying	69.93 (AS) 72.58 (APS)	Elevated the viability of probiotics following 2 h of exposure to pH 2.0 simulated gastric fluid	[140]
Whey protein	Probiotic *Lactiplantibacillus plantarum* KLDS 1.0328	Electrospraying	NR	Maintained stable viable counts at 6.82 log CFU/g throughout 28 days of preservation at refrigeration temperature (4 °C)	[141]
Polyvinyl alcohol, sodium alginate, carboxymethyl cellulose	*Lacticaseibacillus rhamnosus* GG (ATCC 53103)	Electrospinning	82.06	Achieved an encapsulation efficiency of 82.06%	[147]
Polyvinyl alcohol, ethyl cellulose	*Lactiplantibacillus plantarum* 69-2	Electrospinning	NR	Exhibited significantly improved antioxidant capacity and retained over 80% survival following continuous simulated gastrointestinal digestion	[149]
Inulin, whey protein isolate fibrils, carrageenan, hyaluronic acid	*Lactiplantibacillus plantarum* 90	Layer-by-layer assembly	NR	Maintained over 60% encapsulated strain survival under harsh acidic conditions, bile salt environments, and simulated gastrointestinal tract conditions	[155]
Zein, curcumin, sodium carboxymethyl cellulose	*Bifidobacterium longum* CCFM1206, *Pediococcus acidilactici* CCFM6432, *Lactobacillus gasseri* CCFM1346	Layer-by-layer assembly	NR	Maintained 10.72 log CFU/g after 4 weeks of refrigeration, far exceeding free cells (8.23 log CFU/g)	[156]
Tannic acid-Mg^2+^ and casein phosphopeptide	*Lyophilized Saccharomyces boulardii* (code CNCM I-745)	Layer-by-layer assembly	NR	Exhibited favorable stability, high cell viability, and prominent antioxidant activity under simulated gastric and intestinal fluids	[157]
Inulin and tannic acid/Ca^2+^ metal-phenolic networks	*Bifidobacterium lactis*	Layer-by-layer assembly	NR	Reached 66.18% viability for encapsulated *Bifidobacterium lactis* strains after bile salt treatment	[158]
Sodium alginate and soy protein isolate	*Lacticaseibacillus paracasei* ProSci-92	Emulsification	92.17	Reached an encapsulation efficiency of 92.17%	[162]
Alginate	*Bifidobacterium bifidum*, *Bacteroides fragilis*, *Lacticaseibacillus rhamnosus*, and *Streptococcus thermophilus*	3D bioprinting	NR	Exceeded 10^7^ CFU viable bacterial counts, persisting for at least six weeks under both room-temperature and refrigerated storage conditions	[164]
Dextran, alginate, Ca-EDTA, chitosan, Eudragit S100	*Escherichia coli* Nissle 1917	Microfluidics	94.1 ± 0.9	Achieved a viable bacterial survival rate 95.2% during simulated gastrointestinal transit for the Eudragit-chitosan-coated formulation	[166]

## Data Availability

No new data were created or analyzed in this study. Data sharing is not applicable to this article.

## References

[1] FAO/WHO (2002). FAO/WHO Joint Working Group Report on Drafting Guidelines for the Evaluation of Probiotics in Food (30 April 2002 and 1 May 2002).

[2] Tan Z., Tian Z., Duan X., Zhang H., Yu W., Xie Q., Xu D., Ding Y., Ying R., Ma J. (2026). Alleviating Effects of 2′-Fucosyllactose Combined with *Bifidobacterium longum* BB536 on Loperamide-Induced Constipation and Depressive-like Behavior. Food Funct..

[3] Tian Z., Tan Z., Zhang X., Xie Q., Song M., Chen X., Ma J., Ren Q. (2026). 6′-Sialyllactose Combined with *Bifidobacterium animalis* Subsp. *lactis* BB12 Relieves Constipation and Depression-like Behaviors Induced by Loperamide in Mice by Regulating the Gut–Brain Axis. J. Agric. Food Chem..

[4] Sun Q., Yin S., He Y., Cao Y., Jiang C. (2023). Biomaterials and Encapsulation Techniques for Probiotics: Current Status and Future Prospects in Biomedical Applications. Nanomaterials.

[5] Luo Y., De Souza C., Ramachandran M., Wang S., Yi H., Ma Z., Zhang L., Lin K. (2022). Precise Oral Delivery Systems for Probiotics: A Review. J. Control. Release.

[6] Kruk M., Lalowski P., Hoffmann M., Trząskowska M., Jaworska D. (2024). Probiotic Bacteria Survival and Shelf Life of High Fibre Plant Snack—Model Study. Plant Foods Hum. Nutr..

[7] Zhu Y., Lv L., Du B., Zhao M. (2026). Next-Generation Microencapsulation Technologies for Probiotic Protection and Precision Delivery. Microb. Biotechnol..

[8] Gao Y., Wang X., Xue C., Wei Z. (2023). Latest Developments in Food-Grade Delivery Systems for Probiotics: A Systematic Review. Crit. Rev. Food Sci. Nutr..

[9] Xie X., Li Q., Jia L., Yuan H., Guo T., Meng T. (2023). Multishell Colloidosome Platform with Sequential Gastrointestinal Resistance for On-Demand Probiotic Delivery. Adv. Healthc. Mater..

[10] Mohamadzadeh M., Fazeli A., Shojaosadati S.A. (2024). Polysaccharides and Proteins-Based Bionanocomposites for Microencapsulation of Probiotics to Improve Stability and Viability in the Gastrointestinal Tract: A Review. Int. J. Biol. Macromol..

[11] Shu D., Liu Y., Xu J., Yuan Y. (2025). Carrier Design Based on Zein: From the Perspectives of Multi-Molecule Encapsulation, Probiotic Encapsulation, and Application Standards. Curr. Res. Food Sci..

[12] Zhong H., Jiang J., Hussain M., Zhang H., Chen L., Guan R. (2025). The Encapsulation Strategies for Targeted Delivery of Probiotics in Preventing and Treating Colorectal Cancer: A Review. Adv. Sci..

[13] Amiri S., Nezamdoost-Sani N., Mostashari P., McClements D.J., Marszałek K., Mousavi Khaneghah A. (2024). Effect of the Molecular Structure and Mechanical Properties of Plant-Based Hydrogels in Food Systems to Deliver Probiotics: An Updated Review. Crit. Rev. Food Sci. Nutr..

[14] Xie A., Zhao S., Liu Z., Yue X., Shao J., Li M., Li Z. (2023). Polysaccharides, Proteins, and Their Complex as Microencapsulation Carriers for Delivery of Probiotics: A Review on Carrier Types and Encapsulation Techniques. Int. J. Biol. Macromol..

[15] Chen X., Niu H., Chen H., Zhu X., Shen M., Xie J. (2023). Interfacial Behavior of Mesona Chinensis Benth Polysaccharide at the Oil-Water Interface and the Stability of the Emulsion. Food Hydrocoll..

[16] Wang J., Dai T., Lin Y., Jia H., Ji Q., Yuan G. (2022). Polysaccharide-Based High-Strength, Self-Healing and Ultra-Sensitive Wearable Sensors. Ind. Crops Prod..

[17] Gao R., Zhao D., Zhou X., Wan Z., Wang X., Rao H., Liu X., Gao X., Hao J. (2025). Advances in Polysaccharide-Based Biopolymers for Probiotic Encapsulation: From Single Polysaccharides to Composite Systems. Curr. Res. Food Sci..

[18] Tan L.L., Sampathkumar K., Wong J.H., Loo S.C.J. (2020). Divalent Cations Are Antagonistic to Survivability of Freeze-Dried Probiotics Encapsulated in Cross-Linked Alginate. Food Bioprod. Process..

[19] Li X., Tiang M.F., Cui X., Li Y., Wang Z., Zhao L., Takriff M.S., Sajab M.S., Abdul P.M., Ding G. (2024). Precisely Controlled Electrostatically Sprayed Sodium Alginate/Carboxymethyl Chitosan Hydrogel Microbeads as Super-Adsorbent for Adsorption of Cationic Dye. Int. J. Biol. Macromol..

[20] Sun L., Zhao J., Chen W., Wang G. (2025). Electrostatically Sprayed Alginate-Carboxymethyl Chitosan Microgel for Colon-Targeted Melatonin Delivery Ameliorates Ulcerative Colitis. Food Biosci..

[21] Sun R., Lv Z., Wang Y., Gu Y., Sun Y., Zeng X., Gao Z., Zhao X., Yuan Y., Yue T. (2024). Preparation and Characterization of Pectin-Alginate-Based Microbeads Reinforced by Nano Montmorillonite Filler for Probiotics Encapsulation: Improving Viability and Colonic Colonization. Int. J. Biol. Macromol..

[22] Zhang Z., Huang Y., Wang R., Dong R., Li T., Gu Q., Li P. (2024). Utilizing Chitosan and Pullulan for the Encapsulation of *Lactiplantibacillus plantarum* ZJ316 to Enhance Its Vitality in the Gastrointestinal Tract. Int. J. Biol. Macromol..

[23] Liu R., Ci X., Liu L., Wang X., Rifky M., Liu R., Sui W., Wu T., Zhang M. (2024). Chitosan Entrapping of Sodium Alginate/Lycium Barbarum Polysaccharide Gels for the Encapsulation, Protection and Delivery of *Lactiplantibacillus plantarum* with Enhanced Viability. Int. J. Biol. Macromol..

[24] Chen B., Li W., Jiang X., Huang Z., Lin L., Lin X., He Z., Lin X. (2024). Entrapment of Multi-Scale Structure of Alginate Beads Stabilized with Cellulose Nanofibrils for Potential Intestinal Delivery of Lactic Acid Bacteria. Int. J. Biol. Macromol..

[25] Wang M., Wang Q., Chen B., Liu J. (2026). A Narrative Review of Nutritional Components, Health Effects, and Disease Prevention Mechanisms of Dairy Products. Front. Public Health.

[26] Jakubowska D., Dąbrowska A.Z., Staniewska K., Kiełczewska K., Przybyłowicz K.E., Żulewska J., Łobacz A. (2024). Health Benefits of Dairy Products’ Consumption—Consumer Point of View. Foods.

[27] López-López A., Moreno-Baquero J.M., Rodríguez-Gómez F., García-García P., Garrido-Fernández A. (2018). Sensory Assessment by Consumers of Traditional and Potentially Probiotic Green Spanish-Style Table Olives. Front. Nutr..

[28] Yuan L., Li Y., Chen M., Xue L., Wang J., Ding Y., Zhang J., Wu S., Ye Q., Zhang S. (2022). Antihypertensive Activity of Milk Fermented by *Lactiplantibacillus plantarum* SR37-3 and SR61-2 in L-NAME-Induced Hypertensive Rats. Foods.

[29] Hurtado-Romero A., Del Toro-Barbosa M., Gradilla-Hernández M.S., Garcia-Amezquita L.E., García-Cayuela T. (2021). Probiotic Properties, Prebiotic Fermentability, and GABA-Producing Capacity of Microorganisms Isolated from Mexican Milk Kefir Grains: A Clustering Evaluation for Functional Dairy Food Applications. Foods.

[30] Ferdousi R., Rouhi M., Mohammadi R., Mortazavian A.M., Khosravi-Darani K., Homayouni Rad A. (2013). Evaluation of Probiotic Survivability in Yogurt Exposed to Cold Chain Interruption. Iran. J. Pharm. Res..

[31] Sibanda T., Marole T.A., Thomashoff U.L., Thantsha M.S., Buys E.M. (2024). Bifidobacterium Species Viability in Dairy-Based Probiotic Foods: Challenges and Innovative Approaches for Accurate Viability Determination and Monitoring of Probiotic Functionality. Front. Microbiol..

[32] Zhang B., Lan W., Xie J. (2022). Chemical Modifications in the Structure of Marine Polysaccharide as Serviceable Food Processing and Preservation Assistant: A Review. Int. J. Biol. Macromol..

[33] Guo H., Qin Q., Chang J.-S., Lee D.-J. (2023). Modified Alginate Materials for Wastewater Treatment: Application Prospects. Bioresour. Technol..

[34] Wang Y.-Q., Zhang Q., Liu J.-C., Yan J.-N., Wang C., Lai B., Zhang L.-C., Wu H.-T. (2024). Construction and Characterization of Alginate/Calcium β-Hydroxy-β-Methylbutyrate Hydrogels: Effect of M/G Ratios and Calcium Ion Concentration. Int. J. Biol. Macromol..

[35] Wu S., Wu J., Yu H., Zhang J., Huang J., Zhou L., Deng L., Li H. (2024). Varying Ratios of M/G in Alginate to Modulate Macrophages Polarization and Its Application for Wound Healing in Diabetic. Int. J. Biol. Macromol..

[36] Wang X., Gao S., Yun S., Zhang M., Peng L., Li Y., Zhou Y. (2022). Microencapsulating Alginate-Based Polymers for Probiotics Delivery Systems and Their Application. Pharmaceuticals.

[37] Zhao Z., Zhao D., Su L., Ding M., Zhang M., He H., Li C. (2024). Encapsulation and Release of Salidroside in Myofibrillar Protein-sodium Alginate Gel: Effects of Different M/G Ratios of Sodium Alginate. Int. J. Biol. Macromol..

[38] Machado A.R., Silva P.M.P., Vicente A.A., Souza-Soares L.A., Pinheiro A.C., Cerqueira M.A. (2022). Alginate Particles for Encapsulation of Phenolic Extract from *Spirulina* sp. LEB-18: Physicochemical Characterization and Assessment of in Vitro Gastrointestinal Behavior. Polymers.

[39] Tordi P., Ridi F., Samorì P., Bonini M. (2025). Cation-alginate Complexes and Their Hydrogels: A Powerful Toolkit for the Development of Next-generation Sustainable Functional Materials. Adv. Funct. Mater..

[40] Phùng T.-T.-T., Đinh H.-N., Ureña M., Oliete B., Denimal E., Dupont S., Beney L., Karbowiak T. (2025). Sodium Alginate as a Promising Encapsulating Material for Extremely-Oxygen Sensitive Probiotics. Food Hydrocoll..

[41] Nguyen N.T., Nguyen B.-P.T., Ho T.-N., Tran C.-N.D., Tran T.-H.H., Nguyen H.-P.H., Nguyen H.-P., Huynh N.-T., Li Y., Phan V.H.G. (2024). Orally Ingestible Medication Utilizing Layered Double Hydroxide Nanoparticles Strengthened Alginate and Hyaluronic Acid-Based Hydrogel Bead for Bowel Disease Management. Int. J. Biol. Macromol..

[42] Zhang L., Tang P., Li S., Wang X., Zong W. (2022). Sodium Alginate-Based Wall Materials Microencapsulated *Lactobacillus plantarum* CICC 20022: Characteristics and Survivability Study. Food Sci. Biotechnol..

[43] Wang K., Zhou W., Zhai J., Du P., Liu L., Li S., Li A. (2024). Diversified Modification Strategies for Sodium Alginate-Based Probiotic-Embedded Gels and Their Potential for Food Applications. Food Sci. Anim. Prod..

[44] Edo G.I., Mafe A.N., Razooqi N.F., Umelo E.C., Gaaz T.S., Isoje E.F., Igbuku U.A., Akpoghelie P.O., Opiti R.A., Essaghah A.E.A. (2025). Advances in Bio-Polymer Coatings for Probiotic Microencapsulation: Chitosan and beyond for Enhanced Stability and Controlled Release. Des. Monomers Polym..

[45] Hu Y., Zhu S., Ye X., Wen Z., Fu H., Zhao J., Zhao M., Li X., Wang Y., Li X. (2024). Oral Delivery of Sodium Alginate/Chitosan Bilayer Microgels Loaded with *Lactobacillus rhamnosus* GG for Targeted Therapy of Ulcerative Colitis. Int. J. Biol. Macromol..

[46] Yi L., Cheng L., Yang Q., Shi K., Han F., Luo W., Duan S. (2024). Source, Extraction, Properties, and Multifunctional Applications of Pectin: A Short Review. Polymers.

[47] Wei X., Xu K., Qin W., Lv S., Guo M. (2024). Hawthorn (*Crataegus pinnatifida*) Berries Ripeness Induced Pectin Diversity: A Comparative Study in Physicochemical Properties, Structure, Function and Fresh-Keeping Potential. Food Chem..

[48] Hu L., Wang C., Bai Y., Khalifa I., Liang X., Zhang H., Jia Y. (2025). Pectin–Polyphenol Interactions: A System with Enhanced Functionalities and Bioavailabilities. Food Chem..

[49] Valladares L., Vio F. (2025). Pectin and Its Beneficial Effect on Health: New Contributions in Research and the Need to Increase Fruits and Vegetables Consumption—A Review. Int. J. Mol. Sci..

[50] Xu D., Zhao X., Mahsa G.C., Ma K., Zhang C., Rui X., Dong M., Li W. (2023). Controlled Release of *Lactiplantibacillus plantarum* by Colon-Targeted Adhesive Pectin Microspheres: Effects of Pectin Methyl Esterification Degrees. Carbohydr. Polym..

[51] Gutierrez-Alvarado K., Chacón-Cerdas R., Starbird-Perez R. (2022). Pectin Microspheres: Synthesis Methods, Properties, and Their Multidisciplinary Applications. Chemistry.

[52] Jin Z., Wang Y. (2026). Recent Progress in Probiotic Encapsulation: Techniques, Characterization and Food Industry Prospects. Foods.

[53] Shanuke D.S., Ranasinghage N.B.D.P., Illippangama A.U., Kulathunga J., Bandara M.D. (2025). Co-encapsulation of Probiotics and Prebiotics: Techniques and Applications in Food Fortification. Food Sci. Nutr..

[54] Kahraman O., Petersen G.E., Fields C. (2025). Valorization of Coffee Pulp: Spray-Dried Hemp Oil Microcapsules Stabilized with Coffee Pectin and Maltodextrin. Sustainability.

[55] Noreen A., Nazli Z.-H., Akram J., Rasul I., Mansha A., Yaqoob N., Iqbal R., Tabasum S., Zuber M., Zia K.M. (2017). Pectins Functionalized Biomaterials; a New Viable Approach for Biomedical Applications: A Review. Int. J. Biol. Macromol..

[56] Wen C., Lin X., Wang J., Liu H., Liu G., Xu X., Zhang J., Liu J. (2025). Protein–Pectin Delivery Carriers for Food Bioactive Ingredients: Preparation, Release Mechanism, and Application. Compr. Rev. Food Sci. Food Saf..

[57] Rao Y., Deng J., Zhang C., Song Y., Liu L. (2025). Probiotics Encapsulated by Calcium Pectin/Chitosan-Calcium Pectin/Sodium Alginate-Pectin-Whey through Biofilm-Based Microencapsulation Strategy and Their Preventive Effects on Ulcerative Colitis. Food Hydrocoll..

[58] Zhang Q., Yang Y., Chen Y., Ban S., Gu S., Li F., Xue M., Sun J., Li X., Tie S. (2025). Optimization of pH-Responsive Microgel for the Co-Delivery of *Weizmannia coagulans* and Procyanidins to Enhance Survival Rate and Tolerance. Food Chem..

[59] Mikusheva N.G., Zorin I.M., Gubarev A.S., Ievlev A.V., Volina O.V., Tsvetkov N.V. (2025). Influence of Reduced Molar Mass of Low-Acyl Gellan Gum on Weak Gel Formation and Rheological Properties. Gels.

[60] Świerczyńska M., Kudzin M.H., Chruściel J.J. (2024). Poly(Lactide)-Based Materials Modified with Biomolecules: A Review. Materials.

[61] González-Cuello R., Hernández-Fernández J., Ortega-Toro R. (2025). Optimization of the Viability of Microencapsulated *Lactobacillus reuteri* in Gellan Gum-Based Composites Using a Box–Behnken Design. J. Compos. Sci..

[62] Shi J., Yu N., Zhou X., He M., Mao S., Zhu X., Zhang Q., Zuo W., Yang J., Zhang X. (2024). Sodium Caseinate/Gellan Gum Emulsion Gels for Zeaxanthin Dipalmitate Delivery: Preparation, Characterization, and Gelation Mechanism. Int. J. Biol. Macromol..

[63] Huang H., Yan W., Tan S., Zhao Y., Dong H., Liao W., Shi P., Yang X., He Q. (2024). Frontier in Gellan Gum-Based Microcapsules Obtained by Emulsification: Core-Shell Structure, Interaction Mechanism, Intervention Strategies. Int. J. Biol. Macromol..

[64] Gomes D., Batista-Silva J.P., Sousa A., Passarinha L.A. (2023). Progress and Opportunities in Gellan Gum-Based Materials: A Review of Preparation, Characterization and Emerging Applications. Carbohydr. Polym..

[65] Abdl Aali R., Al-Sahlany S. (2024). Gellan Gum as a Unique Microbial Polysaccharide: Its Characteristics, Synthesis, and Current Application Trends. Gels.

[66] Thinkohkaew K., Jonjaroen V., Niamsiri N., Panya A., Suppavorasatit I., Potiyaraj P. (2024). Microencapsulation of Probiotics in Chitosan-Coated Alginate/Gellan Gum: Optimization for Viability and Stability Enhancement. Food Hydrocoll..

[67] Najafpour Z., Golmakani M., Moosavi-Nasab M., Hosseini S.M.H., Niakousari M. (2025). Gellan Fluid Gel Embedded in Alginate Bead System: A New System for Encapsulation of *Limosilactobacillus reuteri* and Its Application in Sour Cherry Juice. Food Sci. Nutr..

[68] Tabassum Z., Anand A., Bhanot R., Girdhar M., Kumar A., Mamidi N., Mohan A. (2025). Xanthan Gum-Driven Innovations for Reinventing Food Preservation. Polymers.

[69] Kumar P., Kumar B., Gihar S., Kumar D. (2024). Review on Emerging Trends and Challenges in the Modification of Xanthan Gum for Various Applications. Carbohydr. Res..

[70] You F., Wu Y., Guo Y., Zheng Y. (2025). Rheological Aspects of Xanthan Gum: Governing Factors and Applications in Water-Based Drilling Fluids and Enhanced Oil Recovery. Carbohydr. Polym..

[71] Wu Q., Lu Z., Wang L., Peng S., Wang Z., Qiu Y., Liao Z., Wang Y., Qin X. (2025). Konjac Glucomannan/Xanthan Gum Hydrogels Loaded with Metal-Phenolic Networks Encapsulated Probiotic to Promote Infected Wound Healing. Carbohydr. Polym..

[72] Koh W.Y., Lim X.X., Tan T.-C., Kobun R., Rasti B. (2022). Encapsulated Probiotics: Potential Techniques and Coating Materials for Non-Dairy Food Applications. Appl. Sci..

[73] Hassanisaadi M., Vatankhah M., Kennedy J.F., Rabiei A., Saberi Riseh R. (2025). Advancements in Xanthan Gum: A Macromolecule for Encapsulating Plant Probiotic Bacteria with Enhanced Properties. Carbohydr. Polym..

[74] Shen J., Chen Y., Li X., Zhou X., Ding Y. (2024). Enhanced Probiotic Viability in Innovative Double-Network Emulsion Gels: Synergistic Effects of the Whey Protein Concentrate-Xanthan Gum Complex and κ-Carrageenan. Int. J. Biol. Macromol..

[75] Wang C., Liu X., Zhang Y., Li X., Ge Y., Lan W., Yang L. (2025). Novel Blueberry Leaf Polysaccharide–Xanthan Gum Composite Gels for Curcumin Encapsulation: Enhanced Stability and Controlled Release. Foods.

[76] Hou Y., Zhao J., Yin J., Nie S. (2023). Structural Properties of Bletilla Striata Polysaccharide and the Synergistic Gelation of Polysaccharide and Xanthan Gum. Food Hydrocoll..

[77] Shoukat R., Cappai M., Pilia L., Pia G. (2025). Rice Starch Chemistry, Functional Properties, and Industrial Applications: A Review. Polymers.

[78] Rodrigues L.D.A., Guerrini L.M., Oliveira M.P. (2025). Graft Copolymerization of Bio-Based β-Farnesene and n-Butyl Acrylate onto Modified Anionic Cassava Starch via Emulsifier-Free Emulsion Polymerization for Sustainable Adhesives. Carbohydr. Polym..

[79] Zhiguang C., Haixia Z., Min C., Fayong G., Jing L. (2025). The Fine Structure of Starch: A Review. npj Sci. Food.

[80] Zhao D., Li Z., Xia J., Kang Y., Sun P., Xiao Z., Niu Y. (2023). Research Progress of Starch as Microencapsulated Wall Material. Carbohydr. Polym..

[81] Zhang X., Liang Y., Bai H., Huang Q., Liu D., Ma G., Liu X. (2025). In Situ Cross-Linked Porous Starch Microencapsulation Enhances the Colonization of *Lactobacillus* in Vivo. Foods.

[82] Liu M., Zhang Z., Zhang Z., Zhou S., Li S., Guo Q., Sun J. (2025). Effect of Microencapsulation Using Peanut Protein Concentrate Combined with Resistant Starch and β-Cyclodextrin on the Viability of *Lactobacillus plantarum* Heal 19 during Storage and in Vitro Digestion. Food Chem. X.

[83] He W., Wu F., Hong H. (2025). Extraction, Utilization, Functional Modification, and Application of Cellulose and Its Derivatives. JRM J. Renew. Mater..

[84] Wang Z.-D., Zhang W., Liang T.-X. (2025). Advancements in Oral Delivery Systems for Probiotics Based on Polysaccharides. Polymers.

[85] Zhong Y., Huang W., Zheng Y., Chen T., Liu C. (2024). Alginate-Coated Pomelo Pith Cellulose Matrix for Probiotic Encapsulation and Controlled Release. Int. J. Biol. Macromol..

[86] Batta-Mpouma J., Kandhola G., Kaur J., Foley K., Walters K.B., Kotagiri N., Kim J.-W. (2025). Cellulose Nanocrystal-Based Hydrogel Microspheres Prepared via Electrohydrodynamic Processes for Controlled Release of Bioactive Compounds. Carbohydr. Polym..

[87] Zhu X., Zhi Y., Heng X., Zhou L., Liu C., Zhao Y., Wang Y., Liu J., Huang J. (2024). Optimization of a Gelatin/Carboxymethylcellulose-based Probiotic Microcapsule and Its Application in Preventing Dextran Sodium Sulfate–Induced Colitis in Mice. J. Food Sci..

[88] Li M.-F., Cui H.-L., Lou W.-Y. (2023). Millettia Speciosa Champ Cellulose-Based Hydrogel as a Novel Delivery System for *Lactobacillus paracasei:* Its Relationship to Structure, Encapsulation and Controlled Release. Carbohydr. Polym..

[89] Tofanica B.-M., Mikhailidi A., Fortună M.E., Rotaru R., Ungureanu O.C., Ungureanu E. (2025). Cellulose Nanomaterials: Characterization Methods, Isolation Techniques, and Strategies. Crystals.

[90] Burchett A., Callahan N., Casini T., Reyes A.D.L., Dornhoefer J., Antony Jose S., Menezes P.L. (2026). Nanocellulose Materials: Processing, Properties, and Application. Nanomaterials.

[91] Xu C., Ban Q., Wang W., Hou J., Jiang Z. (2022). Novel Nano-Encapsulated Probiotic Agents: Encapsulate Materials, Delivery, and Encapsulation Systems. J. Control. Release.

[92] Huang X., Liu R., Wang J., Bao Y., Yi H., Wang X., Lu Y. (2024). Preparation and Synbiotic Interaction Mechanism of Microcapsules of *Bifidobacterium animalis* F1–7 and Human Milk Oligosaccharides (HMO). Int. J. Biol. Macromol..

[93] Campagnollo F.B., Soares M.B., Ramos G.L.P.A., Alvarenga V.O., Chaves R.D., Martinez R.C.R., Sant’Ana A.S. (2024). Modeling the Behavior of Probiotic Strains in Foods under Simulated Gastrointestinal Tract Conditions. Food Biosci..

[94] Ayyash M.M., Abdalla A.K., AlKalbani N.S., Baig M.A., Turner M.S., Liu S.-Q., Shah N.P. (2021). Invited Review: Characterization of New Probiotics from Dairy and Nondairy Products—Insights into Acid Tolerance, Bile Metabolism and Tolerance, and Adhesion Capability. J. Dairy Sci..

[95] Min M., Mason S.L., Bennett G.N., Hussain M.A., Bunt C.R. (2019). Viability Assessment of *Bifidobacterium longum* ATCC 15707 on Non-Dairy Foods Using Quantitative Fluorescence Microscopy. J. Microbiol. Methods.

[96] Maukonen J., Alakomi H.-L., Nohynek L., Hallamaa K., Leppämäki S., Mättö J., Saarela M. (2006). Suitability of the Fluorescent Techniques for the Enumeration of Probiotic Bacteria in Commercial Non-Dairy Drinks and in Pharmaceutical Products. Food Res. Int..

[97] Yan K., Zhang Z., Yang X., Liu L., Wang W., Lu Z., Wang D. (2026). Biomimetic Neurostimulation Framework for Probing Bacterial Metabolism with Anisotropic Au@PDA Nanoassemblies. Anal. Chem..

[98] Krasaekoopt W., Bhandari B., Deeth H. (2003). Evaluation of Encapsulation Techniques of Probiotics for Yoghurt. Int. Dairy J..

[99] Rodrigues F.J., Omura M.H., Cedran M.F., Dekker R.F.H., Barbosa-Dekker A.M., Garcia S. (2017). Effect of Natural Polymers on the Survival of *Lactobacillus casei* Encapsulated in Alginate Microspheres. J. Microencapsul..

[100] Heidebach T., Först P., Kulozik U. (2012). Microencapsulation of Probiotic Cells for Food Applications. Crit. Rev. Food Sci. Nutr..

[101] Yang M., Li L., Zhu X., Liang L., Chen J., Cao W., Liu W., Duan X., Ren G., Liu Z. (2024). Microencapsulation of Fish Oil by Spray Drying, Spray Freeze-drying, Freeze-drying, and Microwave Freeze-drying: Microcapsule Characterization and Storage Stability. J. Food Sci..

[102] Wintzheimer S., Luthardt L., Cao K.L.A., Imaz I., Maspoch D., Ogi T., Bück A., Debecker D.P., Faustini M., Mandel K. (2023). Multifunctional, Hybrid Materials Design via Spray-Drying: Much More than Just Drying. Adv. Mater..

[103] D’Amico V., Siepmann F., Siepmann J., Neut C., Denora N., Lopedota A.A. (2026). Microencapsulation of the Probiotic *Bifidobacterium longum* by Spray-Drying: Formulation and Process Optimisation. J. Drug Deliv. Sci. Technol..

[104] Drosou C., Krokida M. (2024). A Comparative Study of Encapsulation of β-Carotene via Spray-Drying and Freeze-Drying Techniques Using Pullulan and Whey Protein Isolate as Wall Material. Foods.

[105] Murga M.L.F., Cabrera G.M., De Valdez G.F., Disalvo A., Seldes A.M. (2000). Influence of Growth Temperature on Cryotolerance and Lipid Composition of *Lactobacillus acidophilus*. J. Appl. Microbiol..

[106] Alfatama M., Shahzad Y., Choukaife H. (2024). Recent Advances of Electrospray Technique for Multiparticulate Preparation: Drug Delivery Applications. Adv. Colloid Interface Sci..

[107] Song S., Li Z., Li J., Liu Y., Li Z., Wang P., Huang J. (2024). Electrospray Nano-Micro Composite Sodium Alginate Microspheres with Shape-Adaptive, Antibacterial, and Angiogenic Abilities for Infected Wound Healing. ACS Appl. Mater. Interfaces.

[108] Wei L., Zhou D., Kang X. (2021). Electrospinning as a Novel Strategy for the Encapsulation of Living Probiotics in Polyvinyl Alcohol/Silk Fibroin. Innov. Food Sci. Emerg. Technol..

[109] Wu Y., Wang Y., Chen F., Wang B. (2024). Loading Rutin on Surfaces by the Layer-by-Layer Assembly Technique to Improve the Oxidation Resistance and Osteogenesis of Titanium Implants in Osteoporotic Rats. Biomed. Mater..

[110] Wang L., Zhong X., Li S., Liu X., Wang K., Cai R., Yue T., Yuan Y., Wang Z. (2024). Probiotics Encapsulated by Gelatin and Hyaluronic Acid via Layer-by-Layer Assembly Technology for Enhanced Viability. Food Hydrocoll..

[111] Agriopoulou S., Smaoui S., Chaari M., Varzakas T., Can Karaca A., Jafari S.M. (2024). Encapsulation of Probiotics within Double/Multiple Layer Beads/Carriers: A Concise Review. Molecules.

[112] Zhu Y., Wang Z., Bai L., Deng J., Zhou Q. (2021). Biomaterial-Based Encapsulated Probiotics for Biomedical Applications: Current Status and Future Perspectives. Mater. Des..

[113] Rashidinejad A., Bahrami A., Rehman A., Rezaei A., Babazadeh A., Singh H., Jafari S.M. (2022). Co-Encapsulation of Probiotics with Prebiotics and Their Application in Functional/Synbiotic Dairy Products. Crit. Rev. Food Sci. Nutr..

[114] Elvira K.S., Gielen F., Tsai S.S.H., Nightingale A.M. (2022). Materials and Methods for Droplet Microfluidic Device Fabrication. Lab Chip.

[115] Mallick A., Quilès F., Francius G., Burgain J., Gaiani C., Scher J., Amara S., Lemaitre C., Marchal P., Alem H. (2022). An Easy and Robust Method of Preparation of Capsules for Delivering Probiotic Bacteria by a 3D Bioprinting. Food Hydrocoll. Health.

[116] Panda P., Mohanty S., Mohapatra R. (2026). Functional Polymer-Based Encapsulation Technologies for Enhanced Probiotic Therapeutics. J. Drug Deliv. Sci. Technol..

[117] Ek P., Gu B.-J., Dey D., Richter J.K., Saunders S.R., Ganjyal G.M. (2024). Cellulose Interferes with the Transformation of Cornstarch during Extrusion Processing. ACS Food Sci. Technol..

[118] Ullah F.S., Saeed M., Shabbir M.A., Israr B. (2025). Microencapsulation of *Lactobacillus acidophilus* LA-832 and *Bifidobacterium longum* BL-101 by Using Sodium Alginate and Chitosan to Improve the Survival in Simulated Gastrointestinal Conditions. J. Food Sci. Technol..

[119] Gheorghita R.E., Lobiuc A., Covasa M., Muresan A.C., Sirbu I.O. (2026). Biopolymer-Based Gel Capsules for Improved Probiotic Delivery. Gels.

[120] Baleshzar S., Sekhavatizadeh S.S. (2026). Preparation and Characterization of Multilayered Microcapsules of *Lacticaseibacillus rhamnosus* Encapsulated With Sodium Alginate, Jujube Mucilage, and Whey Protein Isolate in Goat Milk Dessert. Food Sci. Nutr..

[121] Mehta N., Kumar P., Verma A.K., Umaraw P., Kumar Y., Malav O.P., Sazili A.Q., Domínguez R., Lorenzo J.M. (2022). Microencapsulation as a Noble Technique for the Application of Bioactive Compounds in the Food Industry: A Comprehensive Review. Appl. Sci..

[122] Kirmizigul Peker A., Guney D., Sengun I. (2024). The Use of Both Free and Microencapsulated *Lactiplantibacillus plantarum* and *Pediococcus parvulus* in Cucumber Pickles. Food Bioprocess Technol..

[123] Xu Y., Dong M., Xiao H., Young Quek S., Ogawa Y., Ma G., Zhang C. (2024). Advances in Spray-Dried Probiotic Microcapsules for Targeted Delivery: A Review. Crit. Rev. Food Sci. Nutr..

[124] Zhao W., Nugroho R.W.N., Odelius K., Edlund U., Zhao C., Albertsson A.-C. (2015). In Situ Cross-Linking of Stimuli-Responsive Hemicellulose Microgels during Spray Drying. ACS Appl. Mater. Interfaces.

[125] Santos Monteiro S., Albertina Silva Beserra Y., Miguel Lisboa Oliveira H., Pasquali M. (2020). Production of Probiotic Passion Fruit (Passiflora Edulis Sims f. Flavicarpa Deg.) Drink Using *Lactobacillus reuteri* and Microencapsulation via Spray Drying. Foods.

[126] Yin M., Chen L., Chen M., Yuan Y., Liu F., Zhong F. (2024). Encapsulation of *Lactobacillus rhamnosus* GG in Double Emulsions: Role of Prebiotics in Improving Probiotics Survival during Spray Drying and Storage. Food Hydrocoll..

[127] Yin M., Chen M., Yuan Y., Liu F., Zhong F. (2024). Encapsulation of *Lactobacillus rhamnosus* GG in Whey Protein Isolate-Shortening Oil and Gum Arabic by Complex Coacervation: Enhanced the Viability of Probiotics during Spray Drying and Storage. Food Hydrocoll..

[128] Boontun C., Vatanyoopaisarn S., Phalakornkule C., Domrongpokkaphan V., Thitisak P., Thaveetheptaikul P., Bamrungchue N. (2024). Influence of Protectant for Encapsulation by Freeze-Drying and Spray-Drying Techniques, and Packaging Environments on the Stability of the Probiotic *Bifidobacterium animalis* subsp. *lactis* Strain KMP-H9-01 during Storage. Dry. Technol..

[129] Bustamante M., Oomah B.D., Burgos-Díaz C., Vergara D., Flores L., Shene C. (2025). Viability of Microencapsulated Probiotics in Cross-Linked Alginate Matrices and Chia Seed or Flaxseed Mucilage During Spray-Drying and Storage. Microorganisms.

[130] Regis F., Arsiccio A., Bourlès E., Scutellà B., Pisano R. (2021). Surface Treatment of Glass Vials for Lyophilization: Implications for Vacuum-Induced Surface Freezing. Pharmaceutics.

[131] Treesinchai S., Puttipipatkhachorn S., Pitaksuteepong T., Sungthongjeen S. (2019). Development of Curcumin Floating Beads with Low Density Materials and Solubilizers. J. Drug Deliv. Sci. Technol..

[132] Wang R., E J., Yang Y., Yang Y., He Y., Gong X., Zheng Y., Zhang Q., Wang J. (2024). Trehalose Enhances the Resistance of *Lactiplantibacillus plantarum* LIP-1 to Spray- and Freeze-Drying Treatments by Regulating Amino Acid Metabolism. Food Biosci..

[133] Acosta-Piantini E., Villarán M.C., Martínez Á., Lombraña J.I. (2024). Examining the Effect of Freezing Temperatures on the Survival Rate of Micro-Encapsulated Probiotic *Lactobacillus acidophilus* LA5 Using the Flash Freeze-Drying (FFD) Strategy. Microorganisms.

[134] Fu N., Hao F., Zhang S., Mao H., Lu W., Chen X.D., Wu W.D. (2024). The Survival and Stability of *Lactobacillus rhamnosus* GG as Affected by Particle Formation during Spray Drying and Spray-Freeze Drying. J. Food Eng..

[135] Dehnad D., Emadzadeh B., Ghorani B., Rajabzadeh G., Tucker N., Jafari S.M. (2023). Bioactive-Loaded Nanovesicles Embedded within Electrospun Plant Protein Nanofibers; a Double Encapsulation Technique. Food Hydrocoll..

[136] Zhang Z., Scherer G.W. (2019). Evaluation of Drying Methods by Nitrogen Adsorption. Cem. Concr. Res..

[137] Jia H., Li Y., Tian Y., Li N., Zheng M., Zhang W., Jiang Y., Zhao Q., Man C. (2025). Recent Advances in Electrospray Encapsulation of Probiotics: Influencing Factors, Natural Polymers and Emerging Technologies. Crit. Rev. Food Sci. Nutr..

[138] Mendes A.C., Chronakis I.S. (2021). Electrohydrodynamic Encapsulation of Probiotics: A Review. Food Hydrocoll..

[139] Laina K.T., Drosou C., Frakolaki G., Krokida M. (2025). Advancing Probiotic Delivery in Functional Yogurt: Encapsulation in Prebiotic-Based Matrices. Foods.

[140] Namazifar F., Shafiekhani A., Hosseini F. (2026). Electrospray Encapsulation of *Lactobacillus plantarum* in Alginate-Starch-Pectin Hydrogels for Enhanced Gastric Protection and Intestinal Delivery. Int. J. Biol. Macromol..

[141] Ma J., Tan Z., Wei X., Tian Z., Wang V.Y., Wang E., Xu D. (2025). Electrospray Whey Protein-Polysaccharide Microcapsules Co-Encapsulating *Lactiplantibacillus plantarum* and EGCG: Enhanced Probiotic Viability and Antioxidant Activity. Int. J. Biol. Macromol..

[142] Moreno J.S., Dima P., Chronakis I.S., Mendes A.C. (2021). Electrosprayed Ethyl Cellulose Core-Shell Microcapsules for the Encapsulation of Probiotics. Pharmaceutics.

[143] Dima P., Stubbe P.R., Mendes A.C., Chronakis I.S. (2023). Electric Field Charge Polarity Triggers the Organization and Promotes the Stability of Electrosprayed Probiotic Cells. Food Hydrocoll..

[144] Ibrahim H.M., Klingner A. (2020). A Review on Electrospun Polymeric Nanofibers: Production Parameters and Potential Applications. Polym. Test..

[145] Deng Y., Lu T., Cui J., Keshari Samal S., Xiong R., Huang C. (2021). Bio-Based Electrospun Nanofiber as Building Blocks for a Novel Eco-Friendly Air Filtration Membrane: A Review. Sep. Purif. Technol..

[146] Li Y., Wang D., Lu Y., Li H., Liu Y., Li Y., Li M., Meng Z., Chen L., Wang H. (2023). Manipulating Mechanical Properties of CaTiO_3_: Eu^3+^ Flexible Fiber Membrane by Fiber Design. J. Am. Ceram. Soc..

[147] Nawaz A., Irshad S., Luo X., Qin Z., Walayat N., Khan M.R., Tahir R., Akram N., Abdi G. (2025). Electrospun PVA/Alginate/Cellulose Nanofibers for Probiotic Delivery: Fabrication, Stability, and in Vitro Viability of Probiotics. Carbohydr. Polym. Technol. Appl..

[148] Tripathy S., Srivastav P.P. (2026). Encapsulation of Food Bioactive Compounds Using Electrohydrodynamic Techniques: From Fundamentals to Industrial Applications. Front. Food Sci. Technol..

[149] Tan Z., Wu M., Li B., Jiang Z., Ma J., Hou J. (2025). Improving Probiotic Survivability Using Coaxial Electrospinning: Role of Ethyl Cellulose in Shell. Food Res. Int..

[150] Ma J., Tan Z., Wu M., Tian Z., Xu C., Zhang J., Ma Y., Feng Z., Yu W., Li B. (2024). Co-Encapsulation of Probiotic *Lactiplantibacillus plantarum* and Polyphenol within Novel Polyvinyl Alcohol/Fucoidan Electrospun Nanofibers with Improved Viability and Antioxidation. Int. J. Biol. Macromol..

[151] Feng K., Huangfu L., Liu C., Bonfili L., Xiang Q., Wu H., Bai Y. (2023). Electrospinning and Electrospraying: Emerging Techniques for Probiotic Stabilization and Application. Polymers.

[152] Yu H., Liu W., Li D., Liu C., Feng Z., Jiang B. (2020). Targeting Delivery System for *Lactobacillus plantarum* Based on Functionalized Electrospun Nanofibers. Polymers.

[153] Campbell J., Vikulina A.S. (2020). Layer-By-Layer Assemblies of Biopolymers: Build-Up, Mechanical Stability and Molecular Dynamics. Polymers.

[154] Zhang Z., Zeng J., Groll J., Matsusaki M. (2022). Layer-by-Layer Assembly Methods and Their Biomedical Applications. Biomater. Sci..

[155] Li S., Su W., Zhang Y., Gan W., Liu X., Fan L. (2025). Enhanced Viability of *Lactiplantibacillus plantarun* by Encapsulation with Whey Protein Isolate Fibrils and Polysaccharides through Layer-by-Layer Coating. Food Hydrocoll..

[156] Liu X., Mao B., Tang X., Zhang Q., Zhai Q., Zhao J., Chen W., Cui S. (2026). Encapsulation of Probiotics Based on Electrostatic Interaction and Layer-by-Layer Assembly Method to Enhance Environmental Resistance. Food Hydrocoll..

[157] Qian Z., Zhai Z., Ren M., Cheng Y., Cao M., Wang Y., Dong L., Wang Y., Cao H., Li C. (2025). Multi-Functionalized Probiotics through Layer-by-Layer Coating with Tannic Acid-Mg^2+^ and Casein Phosphopeptide Complexes for Preventing Ulcerative Colitis. Mater. Today Bio.

[158] Wang M., Zhang S., Liu S., Nian L., Wang S., Cao C. (2025). Inulin and Metal-Phenolic Network Single-Cell Encapsulation: Boosting the Tolerance and Intestinal Colonization Capability of *Bifidobacterium lactis*. Food Hydrocoll..

[159] Han M., Lei W., Liang J., Li H., Hou M., Gao Z. (2024). The Single-Cell Modification Strategies for Probiotics Delivery in Inflammatory Bowel Disease: A Review. Carbohydr. Polym..

[160] Díez-Pascual A., Rahdar A. (2022). LbL Nano-Assemblies: A Versatile Tool for Biomedical and Healthcare Applications. Nanomaterials.

[161] Paker E.S., Senel M. (2022). Polyelectrolyte Multilayers Composed of Polyethyleneimine-Grafted Chitosan and Polyacrylic Acid for Controlled-Drug-Delivery Applications. JFB J. Funct. Biomater..

[162] Peng Q., Hu H., Cui X., Wu J., Han W., Dan T. (2025). Encapsulation of *Lacticaseibacillus paracasei* ProSci-92 in Sodium Alginate/Soy Protein Isolate by Endogenous Emulsification: Enhanced Viability and Storage of Probiotics. LWT.

[163] Zhou X., Fang Y., Zhang T., Xiong Z. (2024). Retrospective: Advances and Opportunities of 3D Bioprinting in China over Three Decades. Addit. Manuf. Front..

[164] Flores-Loera F.J., Cantoral-Sánchez A., Carmona-Ramirez L.F., De Los Santos-Hernández I.P., Montaño-Medina S., Cantú-Fernández D., Pérez-Carrillo E., Rodríguez-Rodríguez J., Luna-Aguirre C.M., Sierra-Valdez F.J. (2025). Bioprinting Microbial Harmony: Engineering Spatially Organized Probiotic Ecosystems via Chaotic Bioprinting. Bioprinting.

[165] Wang K., Huang K., Wang L., Lin X., Tan M., Su W. (2024). Microfluidic Strategies for Encapsulation, Protection, and Controlled Delivery of Probiotics. J. Agric. Food Chem..

[166] Yazdani K., Fardindoost S., Hoorfar M. (2026). Microfluidic Fabrication of Enteric-Coated Liquid-Core ATPS Capsules for Colon-Targeted Probiotic Delivery. Food Hydrocoll..

[167] Araujo H.C.S., De Jesus M.S., Sandes R.D.D., Leite Neta M.T.S., Narain N. (2023). Functional Cheeses: Updates on Probiotic Preservation Methods. Fermentation.

[168] Abdul Manan M. (2025). Progress in Probiotic Science: Prospects of Functional Probiotic-based Foods and Beverages. Int. J. Food Sci..

[169] Kumar D., Ganguly S., Tambde S., Khetra Y., Raju P.N., Hogarehalli Mallappa R. (2026). Development and Characterization of Probiotic Processed Cheese. Sustain. Food Technol..

[170] Guerin J., Burgain J., Borges F., Bhandari B., Desobry S., Scher J., Gaiani C. (2017). Use of Imaging Techniques to Identify Efficient Controlled Release Systems of *Lactobacillus rhamnosus* GG during in Vitro Digestion. Food Funct..

[171] Saeed M., Khanam R., Hafeez H., Ahmad Z., Saleem S., Tariq M.R., Safdar W., Waseem M., Ali U., Azam M. (2024). Viability of Free and Alginate–Carrageenan Gum Coated *Lactobacillus acidophilus* and *Lacticaseibacillus casei* in Functional Cottage Cheese. ACS Omega.

[172] Lopes L.A.A., Pimentel T.C., Carvalho R.D.S.F., Madruga M.S., Galvão M.D.S., Bezerra T.K.A., Barão C.E., Magnani M., Stamford T.C.M. (2021). Spreadable Goat Ricotta Cheese Added with *Lactobacillus acidophilus* La-05: Can Microencapsulation Improve the Probiotic Survival and the Quality Parameters?. Food Chem..

[173] Gutiérrez-Álzate K., De Abreu Barreto G., Rekowsky B.S.S., Alencar Pereira Rodrigues L., Otero D.M., Pereira Da Costa M. (2025). Microencapsulation of *Pediococcus pentosaceus* Using Cupuassu Flour-Based as Wall Material: Application and Impact on Petit Suisse Cheese. Food Biosci..

[174] Bankole A.O., Irondi E.A., Awoyale W., Ajani E.O. (2023). Application of Natural and Modified Additives in Yogurt Formulation: Types, Production, and Rheological and Nutraceutical Benefits. Front. Nutr..

[175] Vacca M., Celano G., Nikoloudaki O., Speckmann B., Calabrese F.M., Gobbetti M., De Angelis M. (2025). Metabolic Characterization of Selected Probiotic Consortia during Gluten and Wheat Bread Simulated Digestion. Food Sci. Hum. Wellness.

[176] Nami B., Tofighi M., Molaveisi M., Mahmoodan A., Dehnad D. (2023). Gelatin-Maltodextrin Microcapsules as Carriers of Vitamin D3 Improve Textural Properties of Synbiotic Yogurt and Extend Its Probiotics Survival. Food Biosci..

[177] Li H., Liu T., Yang J., Wang R., Li Y., Feng Y., Liu D., Li H., Yu J. (2021). Effect of a Microencapsulated Synbiotic Product on Microbiology, Microstructure, Textural and Rheological Properties of Stirred Yogurt. LWT.

[178] Kakili Z., Rezaei P.F., Mahdavinia G., Akbari N. (2025). Improved Oral Delivery and Bioavailability of Novel *Lactobacillus* Isolated from Traditional Maragheh County Yogurt by Encapsulating with Alginate and Carboxymethyl Cellulose. Int. J. Biol. Macromol..

[179] Ebrahimi F., Hosseini A.M., Falah F., Dertli E., Tabatabaei-Yazdi F., Mortazavi S.A., Vasiee A. (2026). Survival of Microencapsulated *Limosilactobacillus fermentum* Strain 4-17 in Alginate–Inulin Matrix during Yogurt Storage. Appl. Food Res..

[180] Gao X., Wang S., Mu G., Tuo Y. (2025). Biofilm Microencapsulation Enhances Survival, Health Benefits, and Yogurt Application of *Lactiplantibacillus plantarum* Y42 Probiotics. Food Biosci..

[181] You S., Zhao X., Cui W., Liu H., Hu J. (2026). The Incorporation of Plant-Derived Polysaccharides into Alginate-Based Capsules Improve Probiotic Viabilities during Storage, Gastrointestinal Digestion, and Their Application in Yogurt. Foods.

[182] De Morais L.C., De Oliveira Meira A.C.F., Freitas T.D., Costa F.F., Carlos L.D.A., Botrel D.A., Veríssimo L.A.A., De Resende J.V. (2026). Effect of Probiotic Microparticles Formed by Alginate and Ora-pro-Nobis Mucilage on the Quality of Ice Cream. Food Control.

[183] Liu Q., Du J., Kuang H., Wang Y., Guo T., Liu G. (2026). Metal-Phenolic Network Encapsulation of *Bifidobacterium animalis* subsp. *animalis* BB04: A Facile Single Cell-Encapsulation Strategy for Enhanced Viability and Probiotic Properties of Probiotic in Ice Cream Applications. Food Biosci..

[184] Lisboa H.M., Sarinho A.M., Costa M.E., Medeiros A.B., Monteiro S.S., Pasquali M.A.B. (2025). Probiotic Ice Cream: Formulation Innovations, Stability Challenges, and Future Perspectives. Food Humanit..

[185] Farias T.G.S.D., Ladislau H.F.L., Stamford T.C.M., Medeiros J.A.C., Soares B.L.M., Stamford Arnaud T.M., Stamford T.L.M. (2019). Viabilities of *Lactobacillus rhamnosus* ASCC 290 and *Lactobacillus casei* ATCC 334 (in Free Form or Encapsulated with Calcium Alginate-Chitosan) in Yellow Mombin Ice Cream. LWT.

[186] Kilinc M., İlkilinc G. (2026). The Effect of the Use of Microencapsulated Stabilizers on Textural Rheological and Physicochemical Properties of Ice Cream. Food Sci. Nutr..

[187] Shishir M.R.I., Khan S., Karim N., Bilal M., Hussain S., Saifullah M., Rashwan A.K., Tahir H.E., Mohamed Ahmed I.A., Zhang X. (2026). New Frontiers in Beverage Fortification: Delivery of Functional Ingredients via Micro- and Nanoencapsulation in Dairy and Non-Dairy Beverages. Food Res. Int..

[188] Cao Y., Yin L., Wang M., Li F., Kong B., Liu Q., Sun F., Wang H. (2025). Improving the Stability and Digestive Property of *Bifidobacterium bifidum* Encapsulated in Whey Protein Isolate/Pectin Emulsions. React. Funct. Polym..

[189] Athayde A.J.A.A., Berger L.R.R., De Albuquerque T.M.R., Sampaio K.B., Fernandes K.F.D., Do Nascimento H.M.A., De Oliveira S.P.A., Lopes L.A.A., De Oliveira C.E.V., Da Conceição M.L. (2025). Physiological and Technological Properties of Probiotic *Lacticaseibacillus rhamnosus* GG Encapsulated with Alginate-Chitosan Mixture and Its Incorporation in Whole Milk. Probiotics Antimicrob. Proteins.

[190] Gutiérrez-Álzate K., Beltrán-Cotta L.A., Dos Santos Rekowsky B.S., Cavalheiro C.P., Pereira Da Costa M. (2024). Micro- and Nanoencapsulation of Probiotics: Exploring Their Impact on Animal-Origin Foods. ACS Food Sci. Technol..

[191] D’Amico V., Cavaliere M., Ivone M., Lacassia C., Celano G., Vacca M., La Forgia F.M., Fontana S., De Angelis M., Denora N. (2025). Microencapsulation of Probiotics for Enhanced Stability and Health Benefits in Dairy Functional Foods: A Focus on Pasta Filata Cheese. Pharmaceutics.

[192] Shu D., Zhang T., Dong Y., Xu J., Yuan Y. (2026). Encapsulation of Probiotics to Improve Their Survival: A Focus on *Bifidobacterium animalis* subsp. *lactis* BB-12. Food Chem. X.

[193] Kaushik R., Gaba K., Anand S., Djira G. (2024). Mathematical Evaluation of Population Changes of *Lactobacillus acidophilus* and *Bifidobacterium animalis* ssp. *Lactis* as Free and Encapsulated Cells in Butter. Fermentation.

[194] Naissinger Da Silva M., Tagliapietra B.L., Pivetta F.P., Richards N.S.P.D.S. (2022). Nutritional, Functional and Sensory Profile of Added Butter from *Lactobacillus acidophilus* Encapsulated and Hyposodium Salt. LWT.

[195] Fang R., Chen T., Hu G., Zhu R., Li X., Wu S., Chen K., Shi C., Xiao G. (2025). Characterization and Storage Stability of Microencapsulated *Lactobacillus helveticus* in Sweetened Condensed Milk: Impacts of Wall Material Composition on Product Quality and Probiotic Viability. Food Chem. X.

